# Evidence-based interventions and nurse-sensitive outcomes in district nursing care: A systematic review

**DOI:** 10.1016/j.ijnsa.2021.100053

**Published:** 2021-11-23

**Authors:** J.D. Veldhuizen, T.B. Hafsteinsdóttir, M.C. Mikkers, N. Bleijenberg, M.J. Schuurmans

**Affiliations:** a Research Centre for Healthy and Sustainable Living, Faculty of Health Care, University of Applied Sciences Utrecht, Utrecht, 3584 CS, the Netherlands; b Department of General Practice, Division Julius Center for Health Sciences and Primary Care, University Medical Center Utrecht, Utrecht, 3508 GA, the Netherlands; c Dutch Healthcare Authority (NZa), Utrecht, the Netherlands; d Tilburg School of Economics and Management, Department of Economics, Tilburg, the Netherlands

**Keywords:** Community health nursing, District nursing, Evidence-based nursing, Health care outcome assessment, Systematic review

## Abstract

**Background:**

Measuring nursing interventions and nurse-sensitive outcomes in a standardized manner is essential because it provides insight into the quality of delivered care. However, there is currently no systematic overview of the interventions conducted by district nurses, the evidence for the effects of these interventions, or what nurse-sensitive outcomes should be measured.

**Objective:**

1) To provide an overview of interventions for community-living older people evaluated in district nursing care and evidence for the effects of these interventions and 2) to identify the nurse-sensitive outcomes that are used to evaluate these district nursing care interventions, how these outcomes are measured, and in which patient groups they are applied.

**Design:**

A systematic review of the literature.

**Setting:**

District nursing care.

**Data sources:**

MEDLINE, CINAHL, PsycInfo, and EMBASE.

**Methods:**

Only experimental studies evaluating district nursing care interventions for communkity-living older people were included. A data extraction form was developed to extract the study characteristics and evaluate interventions and nurse-sensitive outcomes. The methodological quality of the included studies was reviewed using the 13-item critical appraisal tool for randomized controlled trials by the Joanna Briggs Institute.

**Results:**

A total of 22 studies were included. The methodological quality of the studies varied, with scores ranging from 6 to 11 on a scale of 0–13. The 22 interventions identified were heterogeneous with respect to intervention components, intervention delivery, and target population. The 44 outcomes identified were grouped into categories following the Nursing Outcome Classification and were measured in various ways and at various times.

**Conclusion:**

This is the first systematic review summarizing the evidence for the effectiveness of nurse-led interventions conducted by district nurses on community-living older people. It is unclear what interventions are effective and what outcomes should be used to substantiate district nursing care effectiveness. Because only studies with experimental designs were included, this analysis may provide an incomplete assessment of the effectiveness of interventions in district nursing care. Therefore, it is highly necessary to produce methodologically strong evidence through research programs focusing on district nursing care.

## Systematic review registration number

PROSPERO (CRD42017058768).

## Tweetable abstract

The evidence for district nursing care interventions and outcomes is scarce and highly heterogeneous. Robust research programs are needed.

## What is already known about the topic?

• Measuring the effects of nursing interventions and nurse-sensitive outcomes in a standardized manner is crucial, as it provides insight into the quality of delivered care.
• There is currently no systematic overview of the interventions conducted by district nurses, the effects of these interventions, and the measured nurse-sensitive outcomes.

## What this paper adds

• This review demonstrates that experimental studies focusing on district nursing interventions are highly heterogeneous concerning the patient population included, intervention components, execution, structure, and outcome measurements.
• It is unclear which interventions are effective and what outcomes should be used to substantiate district nursing care effectiveness.
• With this scarcity of evidence, it is highly necessary to produce methodologically strong evidence of effective district nursing interventions by conducting robust research programs.

## 1 Introduction

Worldwide, the demand for the delivery of all care at home is predicted to increase greatly in the coming decade. This is due to the rapidly growing ageing population in combination with the desire of the majority of older people to continue to live at home as well as the financial incentives and public demands of health insurers to provide care at home (Jarrín et al., 2019; Maybin et al., 2016; United Nations, 2017; World Health Organization, 2015). District nursing services are the key providers of nursing care in the community, in addition to other healthcare professionals, such as general practitioners and other (paramedic) professionals in primary care (Glasper, 2013; Stall et al., 2014). The organization of district nursing care, including its delivery and funding, varies worldwide (Genet et al., 2012; Jarrín et al., 2019; Van Eenoo et al., 2016). In this study, district nursing care was defined as any technical, medical, supportive or rehabilitative nursing care intervention or assistance with personal care for (older) people living at home (Van Eenoo et al., 2016). This definition is in accordance with the definition used for community-care nursing in Europe (Tarricone and Tsouros, 2008; Van Eenoo et al., 2016) and reflects district nursing care in the Netherlands (Maurits, 2019).

Measuring nursing interventions and nurse-sensitive outcomes in a standardized manner is essential and provides insight into the quality of delivered care, which could guide learning and development in district nursing practice (Jarrín et al., 2019; Pringle et al., 2002). To support nurses in providing care to patients, the nursing intervention classification (NIC) provides a comprehensive, research-based, standardized classification of interventions for nurses and other professionals (Butcher et al., 2018). Interventions are defined as “any treatment, based upon clinical judgement and knowledge, that a nurse performs to enhance patient outcomes” (Butcher et al., 2018). The Nursing Outcome Classification (NOC) is a comprehensive, standardized classification of outcomes to evaluate the impact of interventions provided by nurses or other professionals (Moorhead et al., 2018). Patient outcomes are needed to measure the effects of delivered healthcare services on patients' health and wellbeing (Mant, 2001; World Health Organization, 2006). For district nursing care, it is necessary to focus on nurse-sensitive outcomes, which are patient outcomes that are *relevant* to the nurses’ scope and domain of practice and can be *influenced* by nursing input/interventions (Doran, 2011).

There is currently no systematic overview of the interventions conducted by district nurses or the nurse-sensitive outcomes they achieve for patients (Jarrín et al., 2019; Keleher et al., 2009). While the systematic review by Joling et al. (2018) identified 567 quality indicators for older people for community care, only 18 indicators focused on patient outcomes, of which nine were assessed as nurse-sensitive (Veldhuizen et al., 2021). It is unclear what outcomes are used in district nursing research. A study amongst district nursing care professionals from 17 countries identified a pressing need to generate an evidence base for district nursing care and evaluate home care services and outcomes for patients to guide district nursing care (Jarrín et al., 2019). This evidence is needed because district nursing care is a speciality nursing practice requiring specific nursing interventions and competencies (American Nurses Association, 2007; Community Health Nurses of Canada (CHNC), 2019; Department of Health, 2016; Mildon, 2011; Stuurgroep Kwaliteitskader Wijkverpleging, 2018). Because the literature on interventions and nurse-sensitive outcomes for district nursing care is scarce, a thorough systematic review of the literature is needed.

The aims of this review are 1) to provide an overview of interventions for community-living older people evaluated in district nursing care and evidence for the effects of these interventions; and 2) to identify the nurse-sensitive outcomes that are used to evaluate these district nursing care interventions, how these outcomes are measured, and in which patient groups they are applied.

## 2 Methods

An a priori research protocol was written for this systematic review and published in PROSPERO (CRD42017058768). To guide the systematic review, the steps described in the Joanna Briggs Institute Manual for Evidence Synthesis were followed to conduct the review (Lockwood et al., 2017). To guide the reporting of this manuscript, the Preferred Reporting Items of Systematic reviews and Meta-Analyses (PRISMA) was followed (Moher et al., 2009) (SI Appendix 1).

## 3 Design

### 3.1 Search strategy

Studies evaluating the effectiveness of district nursing interventions were identified using a systematic search. The following electronic databases were searched: MEDLINE, CINAHL, PsycInfo, and EMBASE. The search strategy used a combination of key terms related to nurse-led district nursing care interventions for older people (SI Appendix 2). The search strategy was developed with information specialists from the Cochrane Centre Netherlands and the University of Applied Sciences Utrecht. The database searches were conducted on the 12th of February 2020.

### 3.2 Inclusion criteria

Only empirical studies evaluating district nursing care interventions for community-living older people (aged 60+) and interventions conducted in patients with a mean age of 60 or older were included. Following the advice of the Effective Practice and Organization of Care (EPOC) Group from Cochrane, only randomized controlled trials, controlled clinical trials, controlled before-and-after studies, and interrupted time-series studies were included (Cochrane Effective Practice and Organization of Care Group (EPOC), 2002). Studies evaluating district nurse-led interventions were included. Studies reporting on nurses working in general practices or hospitals and studies in which the nurse's role was unclear were excluded. Studies with at least one face-to-face contact between the district nurse and the patient, either in person or via telehealth, were included. Interventions with only remote monitoring were excluded. To be included, at least one of the outcomes used in the studies had to be nurse-sensitive for district nursing care, following the definition by Doran (2011). No limits were applied on the control group or publication date. Findings from multiple articles reporting on the same study (i.e., reports of the same evaluation of an intervention) were combined. All publications that met the inclusion criteria were uploaded into Rayyan, a web application for systematic reviews that offers researchers a dashboard through which to work through the details of their processes while also allowing full transparency for reviewers (Ouzzani et al., 2016).

### 3.3 Study selection

After all publications were added to Rayyan, duplicate studies were removed. Two reviewers independently assessed the titles and abstracts of all potentially relevant studies for inclusion. In Rayyan, the reviewers were able to read the titles and abstract and make a decision to include or exclude the study. The full texts of studies deemed relevant were obtained, and the assessment of inclusion was repeated independently by two reviewers using Microsoft Excel. To guide the screening and selection of studies, an inclusion criteria screening tool was developed and used by both reviewers (SI Appendix 3). Any disagreements on inclusion were resolved by discussion (JDV and TBH). The results of articles that reported the same study were combined. The number of abstracts and papers identified and excluded, along with the reasons for their exclusion, were recorded.

### 3.4 Data extraction

A data extraction form was developed to extract relevant data from the included studies describing the study characteristics, evaluated interventions and outcomes. The study characteristics extracted were the author names, title, year, country, and design of the study. The intervention data extracted were the study population, sample size, description of the intervention, and a control group description. Regarding the outcomes, the name of the outcome, how the outcome was measured, the measurement instrument or data registry used, the time over which the outcome was measured, and the effects that were measured were extracted. The two reviewers initially piloted the data extraction process with two studies. In the next stage, each reviewer independently extracted data from half of the studies. After extraction, both reviewers checked the data extraction of the other reviewer. The data were compared, and differences were resolved by discussion between the two reviewers (JDV and TBH) until agreement was reached.

### 3.5 Critical appraisal of methodological quality

The studies' methodological quality was independently reviewed by two reviewers (JDV and TBH) using the 13-item critical appraisal tool for randomized controlled trials developed by the Joanna Briggs Institute (Tufanaru et al., 2017). The thirteen items were scored as zero if an item was not met or the item was unclear and as one if an item was clearly met. No single approach is considered the best practice for deciding when a study's quality is sufficient (Porritt et al., 2014). Therefore, the total score of the critical appraisals and risks of bias are presented.

### 3.6 Method of data synthesis

Due to the expected heterogeneity of the included studies, a narrative synthesis was performed to describe the studies in terms of study characteristics, evaluated interventions, and reported outcomes and to provide an overall description of the available evidence. Using content analysis, the outcomes and interventions were thematically categorized and presented narratively. The outcomes were organized into the following categories based on the Nursing Outcome Classification, which is one of the most commonly used standardized nursing terminology (Tastan et al., 2014): functional health, physiological health, psychosocial health, health knowledge and behaviour, perceived health, and family health. The categories of death and healthcare utilization were added following previous research (Akpan et al., 2018; Veldhuizen et al., 2021). Healthcare utilization was used instead of costs when both were described. The total costs of healthcare utilization or interventions were not included in the narrative synthesis.

### 3.7 Ethical approval, informed consent and registration

Ethical approval and informed consent were not required since no participants were involved in this systematic review of the literature. An a priori research protocol for this systematic review is published in PROSPERO (CRD42017058768).

## 4 Results

### 4.1 *Study selection*

**Fig. 1:**
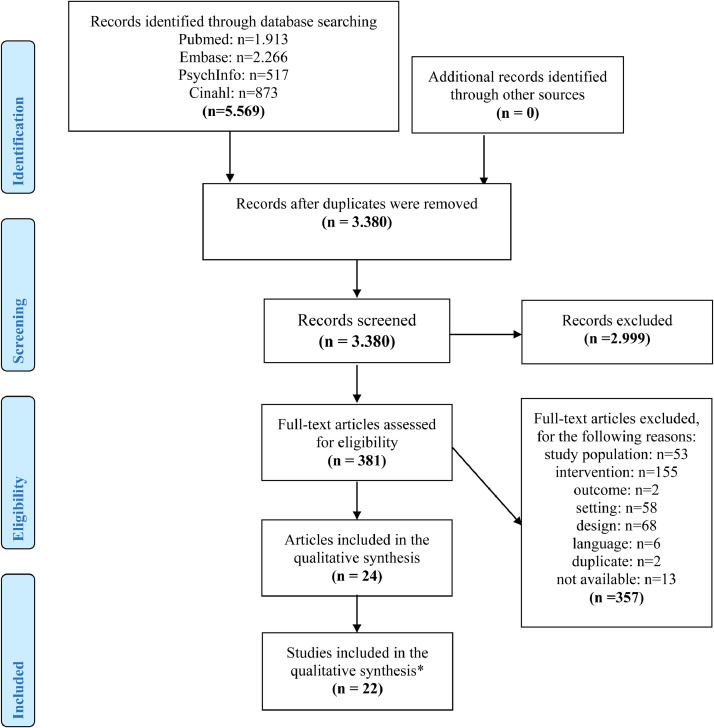
PRISMA flow diagram Notes: * in total, 24 articles were included that described 22 studies. Two studies were described twice in separate articles.

The search resulted in 5569 records. After removing duplicates, 3380 titles and abstracts were screened using the inclusion criteria, and 381 records were retrieved for full-text screening. After the final selection, 22 studies (reported in 24 articles) were included in this systematic review (Fig. 1). In the description of the results below, all studies will be referred to by their reference number between brackets. The reference number and corresponding full reference are provided in Table 1.

**Table 1:** Characteristics of included studies.

Ref #	Author, year, country,	Design, sample patient group	Control group	Intervention	Outcomes measured	Differences in effects between the intervention and control groups	Quality of the study*
1	Toivo et al. (2019), Finland.	Cluster randomized controlled trial, clustered at home care service area (N=5). Older people (65+) (N=188).	Standard home care: care provided by the home care units.	The Coordinated Medication risk Management (CoMM) intervention	•Medication-related outcomes: a) Potentially inappropriate medications; b) excessive use of psychotropics, anticholinergic and serotonergic load; c) clinically significant drug-drug interactions	No statistically significant effects were found for the measures.	6/13
2	Zhu et al. (2018), China.	Randomized controlled trial. People with a diagnosis of hypertension (N=134).	Free annual health check, health education leaflets, and a follow-up with pharmacological treatment.	A nurse-led hypertension management model	• Blood pressure (systolic and diastolic) • Adherence to medication and non-pharmacological behaviours • Self-efficacy • Quality of life • Satisfaction with the care provided	A statistically significant decrease in systolic and diastolic blood pressure and higher satisfaction were observed. No effects were found for other measures.	7/13
3	Buurman et al. (2016), the Netherlands.	Randomized controlled trial.Older people (65+) at risk for functional decline (N=674).	During hospital stay: a comprehensive geriatric assessment was conducted, care was provided and a treatment plan was developed. Multidisciplinary care was provided by a geriatric team. After discharge: no additional care.	Comprehensive Geriatric Assessment and Transitional care bridge program	• Activities of daily living • Mortality status • Cognitive function • Time to unplanned hospital readmission within 6 months • Time to discharge from the nursing home to the community	A statistically significant protective effect was observed for mortality. No effects were found for other measures.	11/13
4	Dorresteijn et al. (2016), the Netherlands.	Randomized controlled trial.Older people (70+) concerned about falling (N=389).	Care as usual: no standard treatment for concerns about falls was available during the study period.	A Matter of Balance (AMB-Home): a home-based, cognitive behavioural program	• Fall-related outcomes: a) concerns about falls; b) avoidance of activity due to concerns about falls; c) number of falls; and d) medical attention received after fall incident • Disability	A statistically significant decrease was observed for indoor falls, disability, concerns about falls and avoidance of activity as a result of concerns about falls. No effects were found for other measures.	11/13
5	Ng and Wong (2018), China; Wong et al. (2016), China.	Randomized controlled trial.People with end-stage heart failure (N=84).	Predischarge palliative care referral consultation and standard discharge planning including a scheduled outpatient palliative care clinic. The control group received two attention control social calls. An unstructured episodic home care service could be arranged for patients upon discharge if needed.	Transitional Care Palliative End-Stage heart failure programme: the Home Palliative heart failure (HPHF) program	• Readmission to hospital • Symptom intensity/burden • Functional status in palliative care • Quality of life • Satisfaction with care • Outcomes related to chronic heart failure (fatigue, dyspnoea, emotional status, mastery) • Caregiver burden	Statistically significant lower readmission to the hospital at 3 months, higher quality of life, higher satisfaction, and lower caregiver burden. Statistically significant lower health complaints were observed for dyspnoea, depression/emotional functioning and mastery at four weeks. No effects were found for other measures.	10/13
6	Suijker et al. (2017), the Netherlands; Suijker et al. (2016), the Netherlands.	Cluster Randomized controlled trial, clustered at general practices (N=24). Older people (70+) at risk for functional decline (N=2283).	Care as usual (not further specified)	Nurse-Led Multifactoral Care to prevent disability in community-living older people	• Disability ((instrumental) activities of daily living) • Health-related quality of life • Quality-adjusted life years • Self-perceived quality of life • Emotional wellbeing • Incidence of falls • All-cause mortality • Healthcare utilization: a) general practitioner consultations; b) general practitioner visits after office hours; c) personal care hours; d) home nursing hours; e) daycare; f) residential care; g) nursing home admission; h) emergency room visits; i) hospital admission	Statistically significant lower general practitioner consultations and costs were observed. Unfavourable higher number of nursing home admission days and hours of personal care and home nursing were observed. No effects were found for other measures.	11/13
7	Sherman et al. (2016), Sweden.	Cluster Randomized controlled trial, clustered at healthcare centre (N=16). Older people (75+) (N=438).	Care as usual (not further specified)	Preventive home care visits by district nurses	• Health index (health and wellbeing) • General health • Health behaviour • Health problems • Knowledge of community/local assistance • Medication use • Satisfaction with intervention	A statistically significant increase in knowledge of community/local assistance was observed. A significant unfavourable higher use of medication was observed. No effects were found on other measures.	7/13
8	Bruce et al. (2016), USA.	Cluster Randomized controlled trial, clustered at nurse teams (N=21). Older people (65+) at risk for depression (N=755).	Enhanced Usual Care: Nurses participated in depression assessment training. They did not receive training and were expected to follow their agencies’ standard procedures for depression.	The Depression CARE for PATients at Home (CAREPATH)	• Hospitalization during intervention • 30-day hospitalization after start of intervention	A statistically significant lower number of hospitalizations was observed. No effect was found for 30-day hospitalization.	6/13
9	Ukawa et al. (2012), Japan.	Randomized controlled trial.Older people (65+) (N=252).	Care as usual: No subjects had any restrictions in receiving medical and formal nursing care.	Functioning Improvement Tool home visit program	• Cognitive functioning	A statistically significant improvement of cognitive function was observed.	8/13
10	Pekmezaris et al. (2012), USA.	Randomized controlled trial.People with heart failure (N=168).	Care as usual: patients were admitted to a certified home healthcare agency following a hospitalization. They were managed via guidelines and standards. Usual care patients received live, face-to-face nursing visits only.	Remote Patient Monitoring	• All-cause hospitalization • Hospital length of stay • Emergency department visit • Healthcare utilization	No statistically significant effects were observed.	9/13
11	Ploeg et al. (2010), Canada.	Randomized controlled trial.Older people (75+) at risk for functional decline (N=719).	Care as usual (not further specified)	Preventive primary care outreach	• Quality-adjusted life-years (disease burden) • Healthcare and social services costs • Functional status • Self-rated health • Mortality	No statistically significant effects were observed.	9/13
12	van Hout et al. (2010), the Netherlands.	Randomized controlled trial.Frail older people (75+) (N=651).	Care as usual: varied from no care at all to regular primary care physician visits to home care involvement.	The preventive home visit program	• Functional status • Disability in (instrumental) activities of daily living • Hospital admittance • Time until nursing home admission • Time until death	No statistically significant effects were observed.	10/13
13	Kwok et al. (2008), China.	Randomized controlled trial.Older people (60 years and older) with chronic heart failure (CHF) (N=105).	Care as usual: Follow-up in hospital outpatient clinics by the same group of designated geriatricians and cardiologists as the intervention group.	Post-discharge community nursing programme	• Functional status • Cognitive function • Psychological state • Handicap • Healthcare utilization: a) community nursing, b) emergency care, c) hospital stay, d) outpatient clinics, 3) readmission	Significantly lower handicap, emergency care and hospital stay rates were observed. No effects were found for other measures.	8/13
14	Bouman et al. (2008), the Netherlands.	Randomized controlled trial.Older people (70+) with poor health status (N=330).	Care as usual: participants could use or apply for all available care within the Dutch healthcare system.	Home visitation program for older people living at home.	• Self-rated health • Functional status ((instrumental) activities of daily living) • Quality of life • Changes in self-reported problems • Health complaints • Depressive complaints • Mental status • Locus of control • Social support • Loneliness • Medication volume and cost • Aids and modifications to the home • Mortality • Use of extramural and institutional care	No statistically significant effects were observed.	9/13
15	Markle-Reid et al. (2006). Canada.	Randomized controlled trial.Older people (75+) (N=288).	Care as usual: using home care services through community-based agencies	Proactive Nursing Health promotion	• Functional status • Mental health (presence of depression) • Perceived social support • Coping style	A statistically significantly greater improvement in mental health functioning (as part of functional status) and reduction in depressive symptom scores were observed. A partial effect was found for perceived social support. No effects were found on other measures	9/13
16	Feldman et al. (2004), USA.	Cluster Randomized controlled trial, clustered at nurse level (N=144). Older people (65+) with chronic heart failure (N=371).	Care as usual (not further specified)	Intervention to Improve Heart Failure Outcomes in Community-Based Home Health Care.	• Healthcare utilization: a) home health nurse visits, b) physician visits, c) inpatient rehospitalization, d) emergency department visits • Quality of life • Satisfaction with the care provided	A statistically significant lower number of home health nurse visits was observed. No effects were found on other measures.	7/13
17	Dougherty et al. (2002), USA.	Randomized controlled trial.Older women (55+) with involuntary urine loss (N=178).	Feedback on information obtained at the baseline visit that neither constituted nor promoted treatment.	Behavioural Management for Continence (BMC)	• Urine loss: a) episodes of urine loss; b) micturition frequency; c) urine loss severity (objective and subjective measure). • Quality of life	Significantly fewer episodes of urine loss (subjective), lower severity of urine loss and higher quality of life were observed.	8/13
18	Hermiz et al. (2002), Australia	Randomized controlled trial.People with chronic obstructive pulmonary disease (COPD) (N=177).	Usual care comprised discharge to general practitioner care with or without specialist follow-up. The discharge did not include routine nursing care or other community follow-up.	Home-Based Care Intervention	• Community nurse visits • Patient satisfaction with care • General practitioner involvement • Admission to emergency department/hospital • Functional status • Knowledge of health • Disease-specific quality of life	The intervention group received statistically significantly more visits from community nurses and displayed greater knowledge and satisfaction. No effects were found on other measures.	6/13
19	Stuck et al. (2000), Switzerland	Randomized controlled trial.Older people (75+) (N=791).	Traditional home care (not further specified)	In-home preventive visits with multidimensional geriatric assessments to prevent disability in community-dwelling older people at low and high risk for nursing home admission.	• Assistance in (instrumental) activities of daily living • Number of permanent admissions to a nursing home • Health care cost and utilization • Affect • Cognitive function • Gait and balance • General health • Number of medications	Partial statistically significant lower assistance in (instrumental) activities of daily living, and higher gait and balance was observed. A partial unfavourable significantly higher number of nursing home admissions was observed. No effects were found on other measures.	8/13
20	van Haastregt et al. (2000), the Netherlands	Randomized controlled trial.Older people (70+) at risk for falls (N=316).	Participants in the usual care group did not receive any special attention or intervention for the prevention of falls and impairments in mobility. The doctors and healthcare staff dealing with the participants were not told which patients were allocated to the usual care group.	Multifactoral home visits	• Falls: 1) number of falls; 2) injurious falls; 3) falls resulting in medical care; 4) fear of falling. • Mobility impairment • Number of physical complaints • Perceived health • Perceived gait problems • Daily activity • Mental health • Social functioning • Loneliness	A significantly lower decline in daily activity and less fear of falling. No effects were found on other measures.	8/13
21	McWilliam et al. (1999), Canada	Randomized controlled trial.Chronically ill older people (65+) (N=298).	Care as usual and attention associated with in-home service, with minimum hours of service equal to the maximum intervention hours.	Home-Based Health Promotion Intervention	• Morale • Self-care agency • Self-esteem • Interpersonal dependency • Locus of authority in decision-making, desire for information • Self-related health, ability to manage health • Rehospitalizations • Quality of life	A statistically significant higher interpersonal dependency, perceived ability to manage health, self-care agency, locus of authority, and quality of life was observed. An unfavourable statistically significant higher desire for information was identified. No effects were found on other measures.	8/13
22	van Rossum et al. (1993), the Netherlands	Randomized controlled trial.Older people (75+) (N=580).	The control group received no home visits. They could use or apply for all the regular services in the area as before.	Preventive home visits for older people.	• Mortality • Self-rated health status • Functional status • Wellbeing: depressive state • Wellbeing: mental state • Healthcare utilization: a) use of community care; b) use of institutional care; c) care expenditure; d) referrals to outpatient clinics.	A significantly lower number of referrals to outpatient clinics was observed. No effects were found on other measures.	9/13

*Notes:* *Methodological quality of the studies, calculated using the 13-item critical appraisal tool for randomized controlled trials by the Joanna Briggs Institute.

### 4.2 *Description of included studies*

The studies were published between 1993 and 2019 and conducted in the Netherlands (3, 4, 6, 12, 14, 20, 22), the United States of America (8, 10, 16, 17), Canada (11, 15, 21), China (2, 5, 13), Australia (18), Finland (1), Japan (9), Switzerland (19), and Sweden (7) (Table 1). Five studies followed a cluster randomized controlled trial design, clustered at the healthcare centre or general practice level (6, 7), home care service level (1), nursing team level (8) or nurse level (16). The remaining 17 studies used a randomized controlled trial design. Measurements were performed between 1 and 36 months after baseline. The sample size ranged from 84 to 2283 participants, and a total of 10,169 older people were involved in the included studies.

### 4.3 *Methodological quality*

Twenty-four articles reported on 22 studies, with two studies being described in two articles (5, 6). The quality scores of the 22 studies ranged from 6 to 11, with a total possible score of 13 (Table 2). The mean and median quality scores of the studies were 8 (IQR: 2,25; Q1-Q3: 6,88–9,13). The weaknesses identified were a lack of blinding and limited description of reliable outcome measurements (i.e., unclear description of the reliability of measurements (Tufanaru et al., 2017)). In seven studies, the outcome assessors were not blinded to treatment assignment (1, 7), or it was unclear whether blinding occurred (2, 16–18, 20). In three studies, the outcomes were measured in a reliable way (4, 5, 21). All studies stated that the outcomes were measured in the same way (i.e., the same instruments and measurement timing were used) between the intervention and control groups.

**Table 2:** Methodological quality.

Published article	Ref#	Q1	Q2	Q3	Q4	Q5	Q6	Q7	Q8	Q9	Q10	Q11	Q12	Q13	Total score*
Toivo et al. (2019)	1	Y	N	N	N	N	N	Y	Y	Y	Y	U	N	Y	6/13
Zhu et al. (2018)	2	Y	U	Y	N	N	U	N	Y	Y	Y	U	Y	Y	7/13
Buurman et al. (2016)	3	Y	Y	Y	Y	N	Y	Y	Y	Y	Y	U	Y	Y	11/13
Dorresteijn et al. (2016)	4	Y	Y	Y	N	N	Y	Y	Y	Y	Y	Y	Y	Y	11/13
Ng and Wong (2018), Wong et al. (2016)	5	Y	Y	Y	N	N	Y	N	Y	Y	Y	Y	Y	Y	10/13
Suijker et al. (2016, 2017)	6	Y	Y	Y	Y	N	Y	Y	Y	Y	Y	U	Y	Y	11/13
Sherman et al. (2016)	7	Y	Y	Y	N	N	N	U	N	Y	Y	U	Y	Y	7/13
Bruce et al. (2016)	8	Y	U	Y	U	N	Y	Y	U	U	Y	U	N	Y	6/13
Ukawa et al. (2012)	9	Y	Y	Y	N	N	Y	Y	Y	U	Y	U	U	Y	8/13
Pekmezaris et al. (2012)	10	Y	Y	Y	N	N	Y	Y	U	Y	Y	U	Y	Y	9/13
Ploeg et al. (2010)	11	Y	Y	Y	N	N	Y	U	Y	Y	Y	U	Y	Y	9/13
van Hout et al. (2010)	12	Y	Y	Y	U	N	Y	Y	Y	Y	Y	U	Y	Y	10/13
Kwok et al. (2008)	13	Y	N	N	N	N	Y	Y	Y	Y	Y	U	Y	Y	8/13
Bouman et al. (2008)	14	Y	Y	Y	U	N	Y	Y	U	Y	Y	U	Y	Y	9/13
Markle-Reid et al. (2006)	15	Y	Y	Y	N	N	Y	Y	N	Y	Y	U	Y	Y	9/13
Feldman et al. (2004)	16	U	U	Y	U	N	U	Y	Y	Y	Y	U	Y	Y	7/13
Dougherty et al. (2002)	17	Y	U	Y	N	N	U	Y	Y	Y	Y	U	Y	Y	8/13
Hermiz et al. (2002)	18	Y	U	Y	U	N	U	Y	N	U	Y	U	Y	Y	6/13
Stuck et al. (2000)	19	Y	U	Y	N	N	Y	U	Y	Y	Y	U	Y	Y	8/13
van Haastregt et al. (2000)	20	Y	U	Y	U	N	U	Y	Y	Y	Y	U	Y	Y	8/13
McWilliam et al. (1999)	21	U	U	Y	U	N	Y	Y	Y	U	Y	Y	Y	Y	8/13
van Rossum et al. (1993)	22	Y	Y	Y	N	N	Y	Y	Y	Y	Y	U	N	Y	9/13
		20/22	12/22	20/22	2/22	0/22	15/22	17/22	16/22	18/22	22/22	3/22	18/22	22/22	

*Notes:* Q1: Was true randomization used for the assignment of participants to treatment groups? Q2: Was allocation to treatment groups concealed?; Q3: Were treatment groups similar at baseline?; Q4: Were participants blind to treatment assignment?; Q5: Were those delivering treatment blind to treatment assignment?; Q6: Were outcome assessors blind to treatment assignment?; Q7: Were treatment groups treated identically other than the intervention of interest?; Q8: Was follow-up complete, and if not, were differences between groups with respect to their follow-up adequately described and analysed?; Q9: Were participants analysed in the groups to which they were randomized?; Q10: Were outcomes measured in the same way in different treatment groups?; Q11: Were outcomes measured in a reliable manner?; Q12: Was an appropriate statistical analysis performed?; Q13: Was the trial design appropriate and any deviations from the standard randomized controlled trial design (individual randomization, parallel groups) accounted for in the conduct and analysis of the trial?’ Y = yes; N = no; U = unclear; * Total score is based on the number of questions answered with ‘yes’.

### 4.4 *Interventions*

A total of 22 interventions were identified (Table 3). None of the included studies evaluated the same intervention. In nine studies, the interventions were conducted following a protocol (2–4, 6, 8, 12, 16, 20, 22), whereas in three studies, only part of the intervention was protocol-dependant (5, 14, 17). The interventions were heterogeneous in the type of patients, intervention components, and delivery.

**Table 3:** Interventions identified and ordered by the target population.

Reference#, reference, name and type of intervention, target population, intervention features, protocol dependency of the intervention	Summary of the intervention	Interventionist, training of interventionist, other professionals involved	Number and duration of visits of calls, total duration of the intervention.
**General population of older people**			
1 – Toivo et al. (2019). Coordinated Medication risk Management (CoMM): A risk management intervention for the general population of older patients (65+) receiving home care. Assessment, triage, referral (meeting with other professionals), implementation of actions. Unclear whether the intervention was protocol-dependant.	In the Coordinated Medication risk Management (CoMM) procedure, the core was a triage that customized medication reviews according to each home care clients’ needs and enhanced use of existing resources. In a drug-related risk screening at home, nurses interviewed their clients using the Drug Related Problem Risk Assessment Tool (DRP-RAT). The drug-related problems needing intervening actions were screened during routine home visits. Findings were reported to the home care team (a leading nurse, nurses and practical nurses), which forwarded the risk screenings to the coordinating pharmacist. Practical nurses also conducted medication reconciliation and compiled medication lists.	Home care practice nurse (n=unclear), Home care nurse (n=unclear), practical nurse (n=unclear) Nurses were trained to screen clinically significant drug-related problems. Pharmacist: consultations were identified based on reports and medication lists Physician: if critical medical concerns were identified, the client's physician was contacted. These consultations took place in collaborative triage meetings.	One home visit for screening and triage meetings. The durations of the visit and triage meetings were unclear. Total duration of the intervention: unclear.
7 – Sherman et al. (2016). Preventive home visit intervention for older people (75+). Assessment, care planning, performing interventions, monitoring, referral. Unclear whether the intervention was protocol-dependant.	During a preventive home visit, the district nurses followed a health dialogue guide. The health dialogue followed the nursing process: assessment of health, planning, diagnosis of health needs, nursing intervention and evaluation of nursing care. If any potential health problems were observed, they were evaluated using various assessment tools. If needed, health aid products were prescribed, medications were checked, and care was coordinated. Information was provided regarding activities in the local community, county council facilities and safety at home. Follow-up contacts were possible if needed.	District nurse (N=35). A one-day course was designed explicitly for district nurses. During the course, they received various materials. No other professionals were involved.	One home visit, with additional follow-up contacts if needed. The home visit was expected to last 60 min. Total duration of the intervention: one visit per patient, with multiple patients spread over 12 months.
9 – Ukawa et al. (2012). Functioning Improvement Tool home visit program for older patients (65+) receiving preventive care at home. Providing guidance to complete a tool. Unclear whether the intervention was protocol-dependant.	During a home visit, the nurse or dental hygienist provided guidance to the patients to complete the Functioning Improvement Tool (FIT), which is a tool for identifying problems in daily life and recording the impressions of their daily tasks. It consisted of six steps: 1) recording activities; 2) recording the reason for daily tasks; 3) categorizing daily tasks into “will” or “duty”; 4) calculating the percentage of daily tasks in each category; 5) calculating a cobweb graph and daily task balance; 6) recording impressions of the daily tasks.	Nurse (not further specified) (n=5), dental hygienist (n=1). The nurses and dental hygienist were trained in the appropriate use of the FIT through lectures and role-playing. No other professionals were involved.	Home visits once a month for three months with a duration of 60 minutes per visit. Total duration of the intervention: three months.
12 – van Hout et al. (2010). The preventive home visit program for frail older people (75+). Assessment, care planning, performing interventions, monitoring, referral. The intervention was protocol-dependant.	The visiting program assessed health risks and care needs using the resident assessment instrument (RAI) home care version. The assessments were entered on laptops, which enabled identification of 30 modifiable health risks. Nurses recommended interventions based on the RAI manual and a nationally issued nursing guideline. Individually tailored care plans were executed. The nurses left a copy of the care plan at a person's home to inform and encourage other visiting health professionals to add notes. The nurses visited a patient to execute and monitor the care plan, evaluate changes in care needs, and adapt the care plan when needed.	Community nurses (n=8) Nurses were trained during a 2-day session. No other professionals were involved. In case of urgent medical matters, the nurses were allowed to consult the primary care physicians.	At least four visits within a year. Additional visits or phone contacts, if necessary. Duration of a visit ranged from 45–75 minutes. Total duration of the intervention: 18 months.
15 – Markle-Reid et al. (2006). Proactive Nursing Health promotion for older people (75+) in need of personal support services. Assessment, education, care planning, performing interventions and referral. Unclear whether the intervention was protocol-dependant.	In the intervention, the participant's resources and environmental supports were bolstered by conducting an initial and ongoing health assessment, identifying and managing risk factors for functional decline, providing health education regarding healthy lifestyles and the management of chronic illnesses, referral to and coordination of community services, building a trusting, supportive and meaningful relationship with the client and his or her caregiver, and providing caregiver support. Factors influencing health were identified and addressed together with clients through the development of a care plan.	Home care registered nurse (n=unclear) The nurses had basic education. Unclear whether nurses were trained with regard to conducting the study. No other professionals were involved.	Home visits or telephone contacts with a duration of ≥10 minutes. The median number of visits was five home visits and one telephone call. The average time per visit was 60 minutes. Total duration of the intervention: 6 months.
19 – Stuck et al. (2000). Disability prevention in community-dwelling older people (75+) at low and high risk for nursing home admission. Assessment, physical examination, problem identification, care planning, monitoring. Unclear whether the intervention was protocol-dependant.	A health nurse obtained medical histories, administered physical examinations, and measured haematocrit and glucose levels in blood samples. Additionally, a comprehensive geriatric assessment was performed, focusing on hearing, vision, nutritional status, oral health, appropriateness of medication use, safety in the home, access to the external environment, and social support. Based on this in-home visit, the nurse prepared a problem list and discussed each case with one of the project team's geriatricians and developed rank-ordered recommendations. In-home follow-up visits were implemented every three months to monitor the implementation of the recommendations, make additional recommendations if new problems were detected and facilitate compliance.	Health nurse (registered nurse with an additional degree in public health nursing) (n=3) The nurses received training regarding physical assessment, gerontology, and performance of preventive home visits before and during the project. Geriatrician: the problems identified by the nurse were discussed with the geriatrician.	In-home visits every 3 months (total of 8 visits). The mean duration of a single visit was 74 minutes. Total duration of the intervention: 2 years.
21 – McWilliam et al. (1999). Home-Based Health Promotion Intervention for chronically ill older people (65+), discharged from the hospital. Reflective dialogue to define needs and action priorities. Unclear whether the intervention was protocol-dependant.	The education intervention focuses on guided reflection by nurses. Through reflective dialogue, individuals were intended to acquire an understanding that altered their expectations, beliefs, values, and perceptions related to their chronic illness experiences. The individualized process focuses on redefining needs and action priorities.	Specialized community home nurses (n=2). Nurses were specially trained (not further specified). No other professionals were involved.	Approximately 12–16 home visits. The duration of a visit was 1 hour. Total duration of the intervention: a maximum of 22 weeks.
22 – van Rossum et al. (1993). Preventive home visits for older people (75+) not receiving home care. Discussing health topics, information provision, advice, referral. The intervention was protocol-dependant. The nurses used a checklist and additional guidelines that were developed to enable them to discuss the various health topics.	During multiple home visits, the nurses discussed health topics, provided information, and gave advice. During the visits, no physical examinations were performed. If necessary, subjects were advised to contact other services. Subjects in the intervention group could also contact the nurse by telephone every day to discuss problems or to ask for an extra visit. Each participant was visited by the same nurse during the entire intervention period, and if subjects became institutionalized, the visits continued as before.	The nurses had been performing in-home care nursing for many years and were employed specifically for the study. Unclear whether the nurses were trained with regard to conducting the study. No other professionals were involved.	Four visits a year for three years with extra visits if necessary. In general, the visits lasted 45 to 60 minutes. Total duration of the intervention: three years.
**Older people with poor health status, at risk for functional decline or falls**			
3 – Buurman et al. (2016). Comprehensive Geriatric assessment and Transitional care bridge program for older patients (65+) at risk for functional decline, discharged from the hospital. Assessment, care planning, performing interventions, monitoring. The intervention was protocol-dependant. Additionally, evidence-based intervention protocols for geriatric conditions were available.	All randomized participants received a systematic, comprehensive geriatric assessment within 48 hours of admission by a geriatric-trained registered nurse. Afterward, the community-care registered nurse was contacted to visit the hospital to receive a personal handover of the assessment, to initiate the personalized care and treatment plan, and to meet with the participant and informal caregiver to discuss their needs. After discharge, the nurse performed medication reconciliation, answered the participant's questions, and completed a needs assessment during a home visit within two days after discharge. If a participant was discharged to a nursing home, the nurse also visited the nursing home. In the following visits, the actions described in the care plan were followed. Geriatric conditions were monitored, and interventions were continued or initiated.	Geriatric-trained registered nurse (n=unclear), community-care registered nurse (n=unclear). Before the start of the intervention, the community-care registered nurse who conducted the transitional care bridge program received ten days of additional training. Geriatrician, geriatric consultation team, the team on the ward in the hospital: conducted the comprehensive geriatric assessment and provided all care needed during a hospital stay.	The assessment was performed in the hospital within 48 hours after admission, a visit by the community-care nurse during admission. Home visits by the community-care nurse at 2, 6, 12, and 24 weeks after discharge. The duration of the visits was unclear. Total duration of the intervention: 24 weeks.
6 – Suijker et al. (2016, 2017). Nurse-Led Multifactoral Care intervention for older patients (70+) at risk for functional decline. Assessment, care planning, performing interventions, care coordination, referral. The intervention was protocol-dependant.	The participants in the intervention group received 1) a systematically administered comprehensive geriatric assessment conducted by the community-care registered nurse; 2) an individually tailored care treatment plan consisting of multifactorial interventions. Diagnostic assessments and interventions were derived from a toolkit containing standardized, evidence-based protocols. Possible interventions were referral to a general practitioner, referral to a paramedic, giving advice, or follow-up visit by the nurse. Subsequently, the nurse discussed the yield of the assessment with the general practitioner; and 3) nurse-led care coordination with multiple follow-up visits.	Community-care registered nurse (n=15). All nurses followed formal 10-day training in providing integrated elderly care in the community before the start of the study. General practitioner: the results of the comprehensive geriatric assessment were discussed with the general practitioner.	One home visit (60 minutes) and between 3–8 additional home visits within 12 months (duration unclear). Total duration of the intervention: 12 months.
11 – Ploeg et al. (2010). Preventive primary care outreach intervention for older patients (75+) at risk for functional decline. Assessment, care planning, health promotion, referral. Unclear whether the intervention was protocol-dependant.	The intervention consisted of a comprehensive initial assessment, collaborative care planning, health promotion, and referral to community health and social support services. An experienced home care nurse delivered the intervention using the resident assessment instrument (RAI) for the home care system. Patient assessments were completed in their homes and triggered new interventions and recommendations at each assessment. Guidelines were used for further assessment and care planning. Referrals were made to various health services. After each visit, the nurses left a card in the home outlining their interventions and any actions required by the patient. The nurses monitored and encouraged patient adherence to their recommendations through follow-up phone calls and home visits.	Home care nurses (n=3). Unclear whether nurses were trained with regard to conducting the study. Family physician: After each home visit, nurses faxed a physician communication form to the patient's family physician. This form outlined the client assessment protocols that were triggered at the visit, nursing actions that were taken to tackle any problems, and areas of follow-up required by the physician.	Three home visits over a year (baseline, after 6 months and after 12 months). Additional home visits or phone calls were possible if necessary. The duration of the visits was unclear. Total duration of the intervention: 12 months.
4 – Dorresteijn et al. (2016). A Matter of Balance (AMB-Home): a cognitive behavioural program for older people (70+) concerned about falling. Problem identification, education about fall-related themes, action planning. The intervention was protocol-dependant.	The intervention consisted of three strategies: 1) identifying and restructuring misconceptions about falls and fall risk; 2) setting realistic personal goals for increasing activity levels and safe behaviour; 3) promoting the uptake of old and new daily life activities that were avoided due to concerns about falling. In the program, seven pre-defined themes of the program were discussed: concerns about falls; thoughts about falling; physical exercise; asserting oneself; overcoming personal barriers; safe behaviour; and managing concerns about falls. Each session was similarly structured with a review of the previous session, a discussion of the main theme, and the formulation of a personalized action plan related to the discussed theme.	Community nurses (n=8) who were qualified in geriatrics and worked at local home care agencies Before the start of the trial, the nurses received a 2-day, mandatory training. No other professionals were involved.	There were seven individual sessions, including three home visits (60, 60 and 75 min, respectively) and four telephone contacts (35 min each). Total duration of the intervention: four months.
20 – van Haastregt et al. (2000). Multifactoral home visits for older people (70+) at risk for falls. Assessment, advice, performing interventions, referral. The nurses followed a structured protocol for home visits.	During the home visits, the older people were screened for several medical, environmental, and behavioural factors potentially influencing falls and mobility. The screening was followed by advice, referrals, and other actions aimed at dealing with the hazards observed.	Community nurse (n=unclear). Unclear whether nurses were trained with regard to conducting the study. No other professionals were involved.	Five home visits. The mean duration per visit was 51 minutes. Total duration of the intervention: 12 months.
14 – Bouman et al. (2008). Home visitation program for older people (70+) with a poor health status living at home. Assessment, advice or referral. The nurses followed a structured protocol to assess health problems and risks via interview. Unclear whether the complete intervention was protocol-dependant.	Participants in the intervention group received a visit approximately every two months, always from the same nurse. To increase adherence, the nurses contacted the older people by telephone 1 to 4 weeks after each visit. During the first visit, the nurses recorded the problems as indicated by the participants. The EasyCare Questionnaire and additional checklists on a variety of topics were then used to detect further problems, which were detected by the nurses using diagnostic instruments. No physical examinations were performed. After the assessment, either advice was given or the older people were referred to professional and community services.	Home nurses (auxiliary community nurses) (n=3) from a local home care organization conducted the visits under the supervision of a public health nurse (community nurse). Unclear whether nurses were trained with regard to conducting the study. General practitioner: They were kept informed at regular intervals. They received an overview of all treated problems for each participant in the intervention group, including the accompanying recommendations and results of the interventions.	The program consisted of 8 visits. The visits lasted between 60 and 90 minutes. Total duration of the intervention: 18 months.
**Disease-specific: chronic heart failure**			
5 – Ng and Wong (2018), Wong et al. (2016). Home Palliative Heart Failure program for end-stage heart failure patients discharged from the hospital to the palliative care team. Assessment, care planning, performing interventions, referral. The interventions in the program were governed by standardized protocols. Unclear whether the complete intervention was protocol-dependant.	The key palliative care components of the Home Palliative Heart Failure program were physical and psychological symptom assessment and management, social support, spiritual and existential aspects of care whenever applicable, setting goals of care, and discussion of treatment preference and end-of-life issues based on patients’ and families’ beliefs and values. The palliative care nurse case managers made referrals to the palliative care physician and other appropriate health services if necessary.	Palliative care nurse case managers (n=4) who were registered nurses with post-registration training in palliative home care and experience in caring for end-stage heart failure patients. Trained volunteers (nursing students) were recruited to support the nurse case managers in the delivery of the intervention. The nurses and volunteers received 18 and 9 h of training, respectively. No other professionals were involved.	One predischarge visit, four visits in the first four weeks. In the subsequent two months, a maintenance intervention dose of monthly home visits supplemented by a social visit and a telephone follow-up by volunteers. The duration of the visit and telephone follow-up was unclear. Total duration of the intervention: 12 weeks.
10 – Pekmezaris et al. (2012). Remote Patient Monitoring, a telehealth intervention for patients with heart failure, discharged from the hospital. Monitoring. Unclear whether the intervention was protocol-dependant.	Both intervention and control group patients were admitted to a certified home healthcare agency following a hospitalization. Both groups were managed via disease management program guidelines and standards of care for heart failure. Patients in remote patient monitoring groups received a combination of live nursing visits and remote patient monitoring visits. The technology utilized closely replicates a face-to-face encounter through two-way video monitoring. With video screens, microphones, and accessories, this technology allows patients and nurses to see each other, speak to each other, and exchange information while in different locations.	Home care nurses (n=unclear). Nurses were trained to teach patients how to manage their conditions through medication, diet, and lifestyle modifications, following a disease management program pathway. No training was available specifically for remote patient monitoring. No other professionals were involved.	A typical visit schedule for patients in the remote patient monitoring group began with one live nursing visit and two remote visits for the first two weeks, followed by an increased frequency of remote visits and a slow tapering of live visits. The duration of the visits was unclear. Total duration of the intervention: 90 days.
13 – Kwok et al. (2008). Post-discharge community nursing programme for older patients (60+) with heart failure discharged from the hospital. Assessment, monitoring and referral. Unclear whether the intervention was protocol-dependant.	The intervention consisted of 1) a visit by a community nurse before discharge from the hospital and 2) a visit within seven days after discharge by a community nurse. During this visit, the nurse checked vital signs and signs for poor control of chronic heart failure. Medications were checked, and if necessary, home and daycare services were arranged. 3) Home visits were performed at weekly intervals for four weeks (not further specified). 4) Home visits were performed monthly after weekly home visits (not further specified). When patients were re-admitted to the hospital, the nurse visited the patient in the hospital to provide background information. Community nurses were available via a telephone hotline during office hours.	Community nurse (n=unclear). Unclear whether nurses were trained with regard to conducting the study. Close collaboration with geriatrician or cardiologist.	Multiple home visits and/or telephone calls. The mean number of visits was 8.8 home visits and/or 5.3 telephone calls. The duration of the visits was unclear. Total duration of the intervention: unclear.
16 – Feldman et al. (2004). Improve Heart Failure Outcomes in Community-Based Home Health Care intervention for older people (65+) with chronic heart failure. Care management, goal setting, education, evaluation The intervention was protocol-dependant.	The intervention consisted of 1) a formal nursing protocol or “Health Outcomes, Management and Evaluation” (“HOME”) Plan, in which the nurse helped the patient with medication, diet and activity recommendations and checked vital signs. The HOME plan outlined twelve specific objectives to be achieved by the nurse within nine visits; 2) a consumer-orientated patient self-care guide; and 3) interactive practitioner training designed to improve nurses’ patient teaching and support skills.	Nurses (not further specified) (n=144). Nurses in the intervention group were trained to augment usual care with the HOME Plan for all of their CHF patients regardless of whether the patient was included in the study. No other professionals were involved.	The interventions consist of nine home visits. The duration of the visits was unclear. Total duration of the intervention: four weeks.
**Disease-specific: other**			
18 – Hermiz et al. (2002). Home Based Care Intervention for patients with chronic obstructive pulmonary disease after discharge from the hospital. Assessment, education, care management, problem identification, care planning, referral, follow-up. Unclear whether the intervention was protocol-dependant.	The intervention comprised two home visits by a community nurse. The first included a detailed assessment of the patient's health status and respiratory function. The nurses provided verbal and written education, advice on the disease and management of care. The nurses identified problem areas and, if indicated, referred patients to other services, such as home care. After the visit, a care plan documenting the problem areas provided education and referral to other services was provided to each patient's general practitioner. At the second home visit, the nurses reviewed patients' progress and the need for further follow-up.	Community nurse (n=unclear). Unclear whether nurses were trained with regard to conducting the study. General practitioner: referral to other services was provided to each patient's general practitioner.	Two home visits: the first within a week after discharge and the second one month later. The duration of the visits was unclear. Total duration of the intervention: one month.
2 – Zhu et al. (2018). A nurse-led hypertension management model for patients with a diagnosis of hypertension. Assessment, care planning, performing interventions, monitoring and referral. The intervention in the study was protocol-dependant. The protocol included information regarding the home visits, telephone follow-ups and referrals.	The intervention consisted of a home visit, a telephone follow-up and referral. 1) The nurse conducted a home visit to patients within three days after recruitment. The patient's knowledge, behaviour, and the status of their identified health problems were assessed. According to the results, the nurses performed relevant interventions. 2) After the home visit, follow-up via telephone calls was conducted biweekly by the nurse. Previous health problems, the current condition of patients, and modifications in their knowledge, behaviour, and status were monitored. The previous behavioural contract was also reviewed and discussed. 3) When the patient reported increased blood pressure, a trained nurse would assess their adherence and/or any current illnesses or living circumstances that may affect their blood pressure. If needed, the patient was referred to other health services	Nurses at the community level (not further specified) (n=4). Nurses were trained during a 36-h pre-intervention training to enhance the nurses’ decision making. General practitioner: If the patient had symptoms that required medication adjustment or a further health check, they were referred to the general practitioner. The general practitioner was responsible for providing (pharmacological) treatment. Researcher: The researcher was in charge of support for the nurses’ decision making and assessment of the quality of care delivered.	One home visit of 60 minutes, biweekly telephone follow-up calls of 10 minutes. Total duration of the intervention: four weeks.
8 – Bruce et al. (2016). The Depression CARE for PATients at Home (CAREPATH), a depression management intervention for older people (65+) at risk for depression. Assessment, care management, goal setting. The intervention was protocol-dependant (the depression care management protocol and CAREPATH protocol).	The intervention guides nurses in managing depression during routine home visits. For individuals who screened positive for depression, nurses assessed depression severity using the 9-item patient health questionnaire, with higher scores indicating severe depression. For beneficiaries with a score of 10 or greater, nurses followed depression care management guidelines during routine visits, including weekly depressive symptom assessment using the patient health questionnaire, care coordination with physicians or specialists, management of side effects and adherence to antidepressant medications, beneficiary and family education, and assistance with setting short-term functional and behavioural goals. For beneficiaries with lower scores, the protocol included education and encouragement, weekly monitoring for 2 weeks, and employing the full protocol when needed.	Home health nurses (not further specified) (n=178). Both the intervention and control groups received depression assessment training. The intervention group also received training in depression management. Physicians, primary clinicians: discussed care coordination.	The intervention was conducted during routine visits. The protocol should be followed weekly or, for patients seen less frequently, at each visit. The duration of the visits was unclear. Total duration of the intervention: unclear.
17 – Dougherty et al. (2002). Behavioural Management for Continence (BMC) intervention for older women (55+) with involuntary urine loss. Performing interventions, training. The bladder training was protocol-dependant. Unclear whether the complete intervention was protocol-dependant	The intervention consisted of three sequenced stages: 1) self-monitoring, 2) bladder training, and 3) pelvic muscle exercise with biofeedback. At the start of the intervention, the nurse and the participant established the woman's goals for continence. The patients decided whether they wanted to begin with self-monitoring or bladder training. After bladder training, the nurse and participant used the bladder diary and goals for continence to decide whether the participant continued pelvic muscle exercise with biofeedback. A re-evaluation of outcome variables and goals was obtained at the end of each phase. If the woman's goals were achieved, the intervention was ended.	Community-based nurses (n=unclear). Unclear whether nurses were trained with regard to conducting the study. No other professionals were involved.	Behavioural management for continence required 20–24 weeks: a) self-monitoring ±2–4 weeks; b) bladder training ±6–8 weeks; and c) pelvic muscle exercise with biofeedback ±12 weeks. It was unclear how many visits were conducted and how long the visits lasted.

*Notes:* The numbers in the first column are the reference numbers of the included studies from Table 1.

### 4.5 Patient groups

Interventions focused on different patient groups, with most studies including older patients in general (1, 7, 9, 12, 15, 19, 21, 22) or older people with a poor health status (14), older people at risk for functional decline (3, 6, 11), older patients at risk for falls (4, 20), or (older) patients with (end-stage) heart failure (5, 10, 13, 16).

### 4.6 Intervention components

In total, 20 of the 22 included interventions consisted of various components. None of the interventions or intervention components were comparable. Similar components amongst the interventions were assessment or problem identification (1–8, 11–14, 18–20); care planning, goal setting, action planning or defining needs and action priorities (2–8,11, 12, 15, 16, 18, 19, 21); referral or triage (1, 2, 5–7, 11, 12–15, 18, 20, 22); regular care interventions, physical examinations, or implementation of actions (e.g., helping a person with medication) (1–3, 5–7, 12, 15, 17, 19, 20); monitoring, evaluation or follow-up (2, 3, 7, 10, 12, 13, 16, 18, 19); education, information provision, health promotion or advice (4, 11, 14–16, 18, 20, 22); care coordination or care management (6, 8, 16, 18); reflective dialogue or health theme discussion (21, 22), and providing guidance or training (9, 17). In total, 18 interventions included three or more of the aforementioned components. The interventions were delivered via home visits (1, 3, 6–10, 14, 16–22) or a combination of home visits and telephone contact (2, 4, 5, 11–13, 15). The number of contact moments via home visits or telephone calls varied between one and sixteen visits. In six interventions, it was possible to have additional contact if needed. The duration of the contact moments ranged from 10–90 minutes.

### 4.7 Interventionists

The nurses delivering the interventions were referred to as home care practice nurses, home care (registered) nurses, (practical) nurses, district nurses, community (home) nurses, home (health) nurses, community-care registered nurses, or palliative care nurse case managers. In total, 413 nurses were involved in the included studies. In nine studies, it was not clear how many nurses were involved (1, 3, 10, 13, 15, 17, 18, 20, 22). A dental hygienist (9) or nursing student (5) conducted the intervention in combination with nurses in two studies. In 10 studies, other healthcare professionals were involved in conducting part of the study (e.g., for conducting a comprehensive assessment; for reference when medical attention was needed; or for discussing identified care needs, care provision or care coordination) (1–3, 6, 8, 11, 13, 14, 18, 19). In 14 studies, the nurses had special training on how to conduct the intervention (1–10, 12, 16, 19, 21).

### 4.8 *Nurse-sensitive outcomes*

In total, 44 nurse-sensitive outcomes were identified, grouped into various categories and measured in various ways at different time points. The identified outcomes were grouped into the following eight categories based on the Nursing Outcome Classification (Moorhead et al., 2018): functional health outcomes (n=5); physiological health outcomes (n=7); psychosocial health outcomes (n=8); health knowledge and behaviour outcomes (n=7); perceived health outcomes (n=6); family health outcomes (n=1); death outcomes (n=1); and healthcare utilization outcomes (n=9) (Table 4). The outcomes measured most often in the studies were quality of life (2, 5, 6, 14, 16–18, 21), activities of daily living (3, 4, 6, 12, 14, 19–21), (self-rated) general health (7, 11, 14, 19–22), functional status (5, 11–13, 15, 22), cognitive functioning (3, 9, 13, 14, 19, 22), time to death or mortality status/rate (3, 6, 11, 12, 14, 22), and satisfaction with provided care (2, 5, 16, 18). With regard to healthcare utilization, the most often measured outcomes were (time to) hospital (re)admission (3, 5, 6, 8, 10, 12–14, 16, 18, 21), community nursing (3, 6, 13, 16, 18, 22), institutionalization (6, 12, 14, 19, 22), and emergency care attendance (6, 10, 13, 14, 16).

**Table 4:** Outcomes used in district nursing care.

**Outcomes**	**Study**	**Patient population**	**Instrument used**	**Time of measurement after baseline**	**Significant effect measured**
**Functional health**					
**Activities of daily living, disability, impairment in mobility, self-care agency**	3, 4, 6, 12, 14, 19, 20, 21	• Older people at risk for functional decline (3, 6) or falls (4, 20) • Frail older people (12) or older people with poor health status (14) • Older people (19, 21)	• Katz index (3, 6) • 18-item GARS (4, 12, 14) • Lawton's multilevel assessment instrument (19) • Mobility control and range scales of the SIP68 (20) • Frenchay activities index (20) • Kearney & Fleisher's self-care agency instrument (21)	• 5–6 months (3, 4, 6, 12, 21) • 12 months (4, 6, 14, 20, 21) • 18 months (6, 12, 14, 20) • 24 months (6, 14) • 36 months (19)	4, 19 (partially), 20 (partially), 21 (at 12 months)
**Functional status**	5, 11, 12, 13, 15, 22	• People with heart failure (5, 13) • Older people (11, 15, 22) • Frail older people (12)	• Palliative Performance Scale (5) • Activities of daily living section of the older Americans resources and services multidimensional functional assessment (11) • COOP-WONCA chart (12) • SF-36 (12, 15, 22) • Six-minute walking test (13)	• 1 month (5) • 2–3 months (5) • 6 months (11, 12, 13, 15) • 12 months (11) • 18 months (12, 22) • 36 months (22)	15 (partially, only for mental health functioning)
**Gait and balance**	19, 20	• Older people (19) • Older people at risk for falls (20)	• Tinetti's fall risk index (19) • Unclear (20)	• 12 months (20) • 18 months (20) • 24 months (19)	19 (partially)
**Self-care adherence**	2	• People with hypertension (2)	• Wong's adherence form (2)	• 3 months (2) • 4 months (2)	
**Handicap**	13	• People with heart failure (13)	• LHS (13)	• 6 months (13)	13

**Physiologic health**					
**Cognitive functioning**	3, 9, 13, 14, 19, 22	• Older people at risk for functional decline (3) • Older people (9, 19, 22) • People with heart failure (13) • Older people with poor health status (14)	• MMSE (3, 9, 14, 19) • AMT (13, 22)	• 3 months (9) • 6 months (3, 13) • 18 months (14, 22) • 24 months (19) • 36 months (22)	9
**Number of medications**	7, 14, 19	• Older people (7, 19) • Older people with poor health status (14)	• Self-developed instrument (7, 14, 19)	• 12 months (7) • 18 months (14) • 24 months (19)	7 (unfavourable)
**Potentially inappropriate medications, excessive use of psychotropic, anticholinergic and serotonergic load, drug-drug interactions**	1	• Older people (1)	• DART (1)	• 12 months (1)	
**Blood pressure (systolic and diastolic)**	2	• People with hypertension (2)	• Calibrated sphygmomanometer and stethoscope (2)	• 3 months (2) • 4 months (2)	2
**Episodes of urine loss**	17	• Older women with urine loss (17)	• Bladder diary (17)	• 6 months (17) • 12 months (17) • 18 months (17) • 24 months (17)	17
**Micturition frequency**	17	• Older women with urine loss (17)	• Bladder diary (17)	• 6 months (17) • 12 months (17) • 18 months (17) • 24 months (17)	
**Urine loss severity in grams**	17	• Older women with urine loss (17)	• Pad test (17) • Self-developed question (17)	• 6 months (17) • 12 months (17) • 18 months (17) • 24 months (17)	17

**Psychosocial health**					
**Mental health, emotional well-being, psychological state**	6, 20,13	• Older people at risk for functional decline (6) or falls (20) • People with heart failure (13)	• SF-36 subscale (20) • SF-36 (6) • GHQ (13)	• 6 months (6, 13) • 12 months (6, 20) • 18 months (6, 20) • 24 months (6)	
**Depressive complaints, affect**	14, 15, 19	• Older people with poor health status (14) • Older people (15, 19)	• GDS (14, 19) • CES-D (15)	• 6 months (15) • 18 months (14) • 24 months (19)	15
**Loneliness**	14, 20	• Older people with poor health status (14) • Older people at risk for falls (20)	• Loneliness scale (14) • Unclear (20)	• 12 months (20) • 18 months (14, 20)	
**Social support**	14, 15	• Older people with poor health status (14) • Older people (15)	• SSL12 (14) • PRQ-85 (15)	• 6 months (15) • 18 months (14)	15 (partially)
**Social functioning**	20	• Older people at risk for falls (20)	• Adjusted version of Donald's social activities battery (20)	• 12 months (20) • 18 months (20)	
**Self-esteem**	21	• Older people (21)	• Rosenberg self-esteem scale (21)	• 5,5 months (21) • 12 months (21)	
**Coping style**	15	• Older people (15)	• Moos’ coping questionnaire (15)	• 6 months (15)	
**Morale**	21	• Older people (21)	• LSI-A (21)	• 5–6 months (21) • 12 months (21)	

**Health knowledge and behaviour**					
**Knowledge about aspects of disease and contact with the local community, desire for information**	7, 18, 21	• Older people (7, 21) • People with COPD (18)	• Self-developed instrument (7, 18) • Locus of authority decision making (21)	• 3 months (18) • 5–6 months (21) • 12 months (7, 12)	7, 18, 21 (unfavourable)
**Self-efficacy, locus of control, locus of authority in decision making**	2, 14, 21	• People with hypertension (2) • Older people with poor health status (14) • Older people (21)	• Short-Form CDSES (2) • Pearlin mastery scale (14) • Beiseckers’ locus of authority in decision-making questionnaire (21)	• 3 months (2) • 4 months (2) • 5–6 month (21) • 12 month (21) • 18 months (14)	21 (at 12 months)
**Number of falls**	4, 6, 20	• Older people (4) • Older people at risk for functional decline (6) or falls (20)	• Self-reported via calendar (4) or questionnaire (6, 20)	• Every month or up to 12 months (4) • 6 months (6) • (Within) 12 months (4) • 12 months (6, 20) • 18 months (6, 20) • 24 months (6)	4 (partially)
**Concerns about falls and avoidance of activity, fear of falling**	4, 20	• Older people (4) • Older people at risk for falls (20)	• 16-item FES-I (4, 20)	• 5 months (4) • 12 months (4, 20) • 18 months (20)	4, 20
**Health behaviour**	7, 18	• Older people (7) • People with COPD (18)	• Self-developed instrument (7, 18)	• 3 months (18) • 12 months (7)	
**Independence to manage health**	21	• Older people (21)	• IDI (21)	• 5–6 months (21) • 12 months (21)	21
**Perceived ability to manage health**	21	• Older people (21)	• SF-36 (21)	• 5–6 months (21) • 12 months (21)	21

**Perceived health**					
**General health (self-rated)**	7, 11, 14, 19, 20, 21, 22	• Older people (7, 11, 19, 21) • Older people with poor health status (14) • Older people at risk for falls (20)	• Health index (7) • Self-developed instrument (7) • Single item from SF-36 (11, 14, 20, 22) • SF-36 (21) • COOP-WONCA chart (19)	• 5–6 months (21) • 12 months (7, 11, 14, 21) • 18 months (14, 22) • 24 months (14, 19) • 36 months (22)	
**Quality of life**	2, 5, 6, 14, 16, 17, 18, 21	• People with hypertension (2), heart failure (5, 16) or COPD (18) • Older people at risk for functional decline (6) • Older people with poor health status (14) • Older women with urine loss (17) • Older people (21)	• LHFQ (16) • SF-36 (2, 14) • SF-20 (14) • McGill quality of life questionnaire (5) • CHQ (5) • EQ-5D (6) • Cantril's Ladder (6) • IIQ (17) • St. George's respiratory questionnaire (18) • Spitzer's QL-Index (21)	• 1 month (5) • 2–3 months (2, 5, 16, 18) • 4 months (2) • 5–6 months (6, 17) • 12 months (6, 17, 21) • 18 months (6, 17) • 24 months (6, 17)	5, 17, 21
**Satisfaction with care provided**	2, 5, 16, 18	• People with hypertension (2), heart failure (5, 16) or COPD (18)	• Modified version of Reeder-Chen's clients satisfaction (16) • Self-developed satisfaction assessment (2, 5) • Unclear (18)	• 1 month (5, 16) • 2–3 months (2, 5, 18) • 4 months (2)	2, 5, 18
**Symptom intensity/burden, health complaints, physical complaints**	5, 14, 20	• People with heart failure (5) • Older people with poor health status (14) • Older people at risk for falls (20)	• ESAS (5) • SCL-90 (14) • Unclear (20)	• 1 month (5) • 2–3 months (5) • 12 months (20) • 18 months (14, 20)	5 (partially)
**Health problems, changes in self-reported problems,**	7, 14	• Older people (7) • Older people with poor health status (14)	• 38 item questionnaire based on VIPS model (7) • Self-developed instrument (14)	• 12 months (7, 14) • 18 months (14) • 24 months (14)	
**Quality-adjusted life-years (QALY)**	11	• Older people (11)	• HUI-mark 3-HRQL utility scores (11)	• 6 months (11) • 12 months (11)	

**Family health**					
**Caregiver burden**	5, 6	• People with heart failure (5) • Older patients at risk for functional decline (6)	• ZBI (5) • CarerQol (6)	• 1 month (5) • 2–3 months (5) • 6 months (6) • 12 months (6) • 18 months (6) • 24 months (6)	5

**Death**					
**Mortality status, time until death, mortality rate, mortality**	3, 6, 11, 12, 14, 22	• Older people at risk for functional decline (3, 6) • Older people (11, 22) • Frail older people (12) or older people with poor health status (12, 14)	• Registry or claims records (3, 6, 11, 12, 14, 22)	• 1 months (3) • 6 months (3, 6, 12) • 12 months (6, 11, 22) • 18 months (6, 12) • (Within) 24 months (6, 14, 22) • (Within) 36 months (22)	3

**Healthcare utilization**					
**Health care utilization: (time to) hospital (re)admission (in days)**	3, 5, 6, 8, 10, 12, 13, 14, 16, 18, 21	• Older people at risk for functional decline (3, 6) or depression (8) • People with heart failure (5, 10, 13, 16) or COPD (18) • Frail older people (12) or older people with poor health status (14) • Older people (21)	• Registry or claims records (3, 5, 6, 8, 10, 12, 13, 14, 16, 18, 21)	• (Within) 1 month (5, 8, 10) • (Within) 2–3 months (5, 8, 10, 16, 18) • (Within) 5–6 months (3, 6, 12, 21) • 12 months (6, 12) • 18 months (6, 12) • (Within) 24 months (6, 14)	5 (within 3 months) 8 (within 2 months)
**Healthcare utilization: (time to) community nursing**	3, 6, 13,16, 18, 22	• Older people at risk for functional decline (3, 6) • People with chronic heart failure (13, 16) or COPD (18) • Older people (22)	• Registry or claims records (3, 6, 13, 16, 18)	• 3 months (16, 18) • (Within) 6 months (3, 6, 13) • 12 months (6) • 18 months (6) • 24 months (6)	6 (unfavourable), 16, 18 (unfavourable)
**Health care utilization: (time to) institutionalization to nursing home / care home**	6, 12, 14, 19, 22	• Older people at risk for functional decline (6) • Frail older people(12) or older people with poor health status (14) • Older people (19, 22)	• Registry or claims records (6, 12, 14, 19, 22)	• 6 months (6, 12) • 12 months (6) • 18 months (6, 12) • (Within) 24 months (6, 14) • (Within) 36 months (19, 22)	6 (unfavourable), 19 (partially and unfavourable)
**Healthcare utilization: physician visits during and after working hours**	6, 14, 16, 18	• Older people at risk for functional decline (6) • Older people with chronic heart failure (16) or COPD (18) • Older people with poor health status (14)	• Registry or claims records (6, 14, 16, 18)	• 3 months (16, 18) • 6 months (6) • 12 months (6) • 18 months (6) • (Within) 24 months (6, 14)	6
**Healthcare utilization: emergency care attendance**	6, 10, 13,14, 16	• Older people at risk for functional decline (6) • People with heart failure (10, 13, 16) • Older people with poor health status (14)	• Registry or claims records (6, 10, 13, 14, 16)	• (Within) 1 months (10) • (Within) 3 months (10, 16) • 6 months (6, 13) • 12 months (6) • 18 months (6) • (Within) 24 months (6, 14)	13
**Health care utilization: number of days in hospital wards, hospital stay**	10, 13, 14	• People with heart failure (10, 13) • Older people with poor health status (14)	• Registry or claims records (10, 13, 14)	• (Within) 1 months (10) • (Within) 3 months (10) • 6 months (13) • (Within) 24 months (14)	13
**Health care utilization: referral to outpatient clinics**	13, 14, 22	• People with heart failure (13) • Older people with poor health status (14) • Older people (22)	• Self-registered by patient (13, 14, 22)	• 6 months (13) • (Within) 24 months (14) • (Within) 36 months (22)	22
**Healthcare utilization: physiotherapy contacts**	22	• Older people (22)	• Registry or claims records (22)	• (Within) 36 months (22)	
**Aids and modifications to the home**	14	• Older people with poor health status (14)	• Self-developed questionnaire (14)	• (Within) 24 months (14)	

*Notes:* The numbers in each column are the reference numbers of the included studies from table 1. GARS: Groningen Activity Restriction Scale; SIP68: sickness impact profile short generic version; COOP/WONCA: The Dartmouth Corporation Functional Health Assessment Charts/World Organization of Family Doctors; SF-36: The Medical Outcomes Study 36-item Short-form Health Survey; FES-I: Falls Efficacy Scale-International; VIPS: wellbeing, integrity, prevention and safety; LHS: London Handicap Scale; ESAS: The Edmonton Symptom Assessment Scale; SCL-90: symptom checklist; MMSE: Mini-mental State Examination; AMT: Abbreviated mental test; GHQ: General health questionnaire; GDS: Geriatric depression scale; CES-D: centre for epidemiological studies depression scale; LSI-A: Life Satisfaction Index-version A; CDSES: Chronic Disease Self-Efficacy Scale; ZBI: Zarit Burden Interview; CarerQol: Caregiver quality of life; SSL12: social support list of interactions; PRQ-85: Personal Resource Questionnaire; IDI: Interpersonal dependency inventory; DART: Drug Related Problem Risk Assessment Tool; LHFQ: The Minnesota Living with Heart Failure Questionnaire; CHQ: chronic heart failure questionnaire; EQ-5D: European Quality of Life-5 Dimension; SF-20: Medical Outcomes Study 20-item Short Form Survey Social functioning score; IIQ: Incontinence impact questionnaire; QL-Index: Quality of life index; HUI-Mark3-HRQL: Health Utilities Index Mark 3 health related quality of life.

The outcomes were measured using various instruments. The instruments used in more than two studies were the Groningen Activities Restriction Scale to measure activities of daily living (4, 12, 14); Short Form-36 to measure functional status (12, 15, 22), mental health (6, 20), general health (21) and quality of life (2, 14); single item Short Form-36 to measure general health (11, 13, 20, 22); and the Mini-Mental State Examination to measure cognitive functioning (3, 9, 14, 19). In nine studies, self-developed instruments were used (2, 5–7, 14, 17–20). Data registry or claim records were used in 13 studies to measure healthcare utilization (3, 5, 6, 8, 10, 12–14, 16, 18, 19, 21, 22). Outcomes were measured at various time points, ranging from 1 to 36 months after baseline.

Statistically significant effects of the interventions were found in 27 of the 44 outcomes. Given the variation in the interventions and measured outcomes and to avoid misinterpretation, no effect sizes are provided. Favourable positive statistically significant effects were identified in 16 studies. In seven outcomes, the effect was partial, i.e., the effect was measured within groups instead of between groups or the effect was present at one but not all time points. The outcomes with positive (partial) statistical significance in two or more studies were activities of daily living (4, 19–21), concerns regarding falls (4, 20), knowledge of disease and healthcare (7, 18), hospital readmission (5, 8), quality of life (5, 17, 21), and satisfaction with the care provided (2, 5, 18). For four outcomes, the effect was unfavourable, i.e., the intervention had a negative statistically significant effect on the outcome; specifically, the participant in the intervention group had higher healthcare utilization regarding home nursing (6, 18) and nursing home admissions (6, 19) and less knowledge of aspects of the disease (21) or used more medications (7) than those in the control group.

## 5 Discussion

This is the first systematic review providing an overview of nurse-led interventions conducted by district nurses for community-living older people. A total of 22 randomized controlled trials were identified and described in 24 articles. The studies were highly heterogeneous in methodological quality, the patient population on which the intervention focused, intervention components, and outcome measurements. Therefore, based on the results of this review, it is unclear what interventions are effective for whom and what nurse-sensitive outcomes can be used to show the value of district nursing care.

Our first aim of the review was to provide an overview of interventions evaluated in district nursing care and their effects. The included studies focused on the general population of older people (n=14) and older people with heart failure (n=4) or another specific problem or disease (n=4). This diversity in patient populations reflects district nursing care settings, where nurses perform a wide range of clinical interventions and fulfil a specialist-generalist role in providing care (Scotland's Chief Nursing Officer Directorate, 2017). This underlines that district nursing care is a speciality nursing practice requiring specific nursing interventions and competencies.

The nurses in charge of the interventions had a wide range of positions, roles and job titles (e.g. home care (practice) nurses, district nurses, community (home) nurses, home (health) nurses, or (palliative care) nurse case managers). The studies do not clearly describe the roles or educational levels required for the nurses involved in the intervention. Therefore, it is unclear whether there were differences in the tasks and responsibilities of the nurses, making comparisons complicated. The literature shows that the organization of health and social services, including district nursing care, differs both between and within European countries (Genet et al., 2011). While this variation is needed and inevitable, it is necessary to be transparent about the roles, tasks and responsibilities of those conducting the intervention in district nursing research.

Variation in healthcare interventions is common. Most health care interventions are complex, i.e., include several components with possible interactions, leading to a range of potential and variable outcomes (Richards and Hallberg, 2015). There are many challenges in reviewing complex health interventions (Richards and Hallberg, 2015): it involves variations in intervention doses and patient characteristics, interactions between the intervention and context, and various measures of the same construct and outcomes (Pigott and Shepperd, 2013; Richards and Hallberg, 2015). Following the study by Pigott and Shepperd (2013), the heterogeneity of the studies included in this review was investigated. While some studies made similar comparisons, such as comparing district nursing care to a new intervention or with no care, the intervention components, dosage and delivery of the individual interventions were diverse. None of the intervention components were sufficiently comparable, rendering synthesis of the results using meta-analyses impossible.

Based on the statistically significant effects identified, no distinctive features between the interventions with and without effects were identified. Some of the effects were found only within groups instead of between groups, leading to possible overestimation of the outcome. This had also been identified by a review evaluating the effects of fundamental nursing care interventions, which showed frequent attempts to overestimate the outcomes of studies by claiming positive effects based on within-group effects rather than between-group effects (Richards et al., 2018). Ultimately, the authors decided not to draw any conclusions regarding the effectiveness of the interventions.

The second aim of this review was to identify nurse-sensitive outcomes that are used in studies evaluating district nursing care interventions. The 44 outcomes identified mainly focused on functional health, perceived health, and healthcare utilization. Of the 44 outcomes, 20 were nurse-sensitive, as identified by a Delphi study regarding nurse-sensitive outcomes in district nursing care (Veldhuizen et al., 2021). In contrast, three outcomes were not nurse-sensitive (mortality status, knowledge of the patient, and polypharmacy), and for eight outcomes, it was unclear if the outcomes were nurse-sensitive (SI Appendix 4). The outcomes with favourable (partial) statistical significance were activities of daily living, concerns about falls, knowledge of disease and healthcare, hospital readmission, quality of life and satisfaction with the care provided. These outcomes are potentially most useful for measuring the effect of district nursing interventions. The outcomes were measured in various ways at various time points using a variety of instruments. Therefore, it is currently unclear how these nurse-sensitive outcomes should be used to measure the quality of delivered district nursing care. The quality of the description of outcome measurements was limited in 19 studies. This may threaten the validity of statistical inferences on the existence and magnitude of the effect determined by the treatment (Tufanaru et al., 2017). The reliability of the outcome measurements being unclear or not described could be why only weak effects were identified in the studies.

### 5.1 *Implications for practice and further research*

This review shows that evidence for district nursing care interventions is scarce. This underlines the conclusion by Jarrín et al. (2019), emphasizing the pressing need to develop an evidence base for district nursing care. A first step in developing this evidence base is to pay attention to the methodological quality of the conducted studies. In this review, only a small number of randomized controlled trials were identified. Conducting experimental work through effective research programs focusing on the effects of interventions on outcomes is strongly encouraged (Melnyk, 2012; Richards et al., 2014). For nursing research in general, Richards et al. concluded that less than 10% of articles reported in nursing journals are randomized controlled trials (Richards et al., 2018). When interested in the effectiveness of interventions, more attention should be given to setting up intervention trials with experimental designs such as randomized controlled trials, interrupted time series, or a stepped-wedge design (Richards and Hallberg, 2015). We are, however, aware of the challenges researchers testing (district) nursing interventions face, such as difficulties with randomization. When it is not possible to conduct experimental studies, other study designs and statistical methods could be used to examine the effectiveness of interventions (e.g., causal inference in quasi- and nonexperimental studies). It would be valuable to conduct a review of studies investigating the effectiveness of interventions using other designs than those used in the present review. Additionally, it would be relevant to provide insight into other studies conducted in district nursing care (e.g. the experiences with or feasibility of interventions in district nursing care using qualitative or mixed-methods approaches) to provide insight into all evidence available for district nursing care.

In future research, more attention should be given to the reporting of studies. For complex interventions specifically, the criteria for reporting the development and evaluation of complex interventions in healthcare (CReDECI) should be followed (Möhler et al., 2012). It is essential to provide a thorough description of the outcome measurements, as this was the most critical methodological weakness in the included studies. Additionally, a more detailed and transparent description of who delivers what care, including a description of the roles, tasks and responsibilities, is needed to enhance replication. Also, this study shows great variation in how the outcomes were measured. It is important to measure nurse-sensitive outcomes in a systematic, standardized manner to ensure good transparency of the quality of the care delivered. With this, it is possible to provide guidance in quality monitoring and improve district nursing care quality (Pringle et al., 2002). To conclude, a systematic research program guided by a strong theoretical foundation and focusing on interventions and nurse-sensitive outcomes is needed to produce methodologically strong evidence for district nursing care that is reliable, replicable and robust.

### 5.2 *Strengths and limitations*

This is the first systematic review focusing on nurse-led interventions for community-living older people conducted by district nurses. A strength of this study was that it was conducted systematically following the Joanna Briggs Institute Manual for Evidence Synthesis (Tufanaru et al., 2017) and advice from information specialists from the Cochrane Centre Netherlands and the University of Applied Sciences Utrecht. Reporting was guided using the Preferred Reporting Items of Systematic reviews and Meta-Analyses (PRISMA) (Moher et al., 2009). All steps of this review were conducted independently by two reviewers, minimizing selection bias.

To appreciate the findings of this review, some limitations need to be considered. First, although only studies with experimental designs were included in this review, this may potentially have led to missed interventions. In this study, we followed the advice of the Cochrane Effective Practice and Organization of Care (EPOC) group by including only randomized controlled trials, controlled clinical trials, controlled before-and-after studies, and interrupted time-series studies (Cochrane Effective Practice and Organization of Care Group (EPOC), 2002). However, studies with other designs, including quasi- and nonexperimental designs with rigorous statistical methods, could potentially provide evidence for the effectiveness of district nursing care. Second, it is possible that interventions were missed because the review focused solely on nurse-led interventions conducted by district nurses in the community. While various job titles for district nurses were included in the search strategy, it is possible that studies were missed due to other job titles being used. This was minimized by building the search strategy in collaboration with information specialists. Also, excluding studies conducted in other settings that could be potentially relevant for district nursing care could have led to an incomplete picture. Third, it was impossible to pool the data into a meta-analysis or synthesis; therefore, only a narrative synthesis was conducted.

## 6 *Conclusions*

This review shows that the evidence for district nursing care interventions following an experimental design is scarce and highly heterogeneous. None of the included studies evaluated the same intervention, and the studies varied in the type of patients, intervention components, and outcome measures, which complicated the comparison of studies. Therefore, evidence regarding the effects of district nursing care interventions is inconclusive. Additionally, it is unclear what outcomes can be used to demonstrate the value of district nursing care. There is a pressing need to produce methodologically strong evidence that is reliable, replicable and robust. Research programs guided by theory and focusing on interventions and nurse-sensitive outcomes in district nursing care are highly needed. It is important to measure nurse-sensitive outcomes in a standardized manner to provide insight into the quality of delivered care and to guide monitoring and improve the quality of district nursing care.

## Author contributions

**JDV:** Conceptualization, methodology, formal analysis, investigation, data curation, writing: original draft, writing: review & editing, visualization, project administration. **TBH:** methodology, formal analysis, investigation, data curation, writing: review & editing, supervision. **MCM**: conceptualization, methodology, writing: review, supervision. **MJS:** conceptualization, methodology, writing: review, supervision. **NB:** conceptualization, methodology, writing: review, supervision.

## Declaration of Competing Interest

None.

## Appendix Supplementary materials

Image, application 1
