# Supplementary material for: Evidence-based interventions and nurse-sensitive outcomes in district nursing care: A systematic review
Source: Int J Nurs Stud Adv. 2021 Nov 23;3:100053. doi: 10.1016/j.ijnsa.2021.100053 (PMC11080545; doi:10.1016/j.ijnsa.2021.100053)
Supplement: Supplementary file 1 [file mmc1.docx]

**Supporting Information**

**Appendix 1**

| **Section/topic** | **#** | **Checklist item** | **Reported on page #** |
| --- | --- | --- | --- |
| **TITLE** | | |  |
| Title | 1 | Identify the report as a systematic review, meta-analysis, or both. | 1 |
| **ABSTRACT** | | |  |
| Structured summary | 2 | Provide a structured summary including, as applicable: background; objectives; data sources; study eligibility criteria, participants, and interventions; study appraisal and synthesis methods; results; limitations; conclusions and implications of key findings; systematic review registration number. | 1 |
| **INTRODUCTION** | | |  |
| Rationale | 3 | Describe the rationale for the review in the context of what is already known. | 4-6 |
| Objectives | 4 | Provide an explicit statement of questions being addressed with reference to participants, interventions, comparisons, outcomes, and study design (PICOS). | 6 |
| **METHODS** | | |  |
| Protocol and registration | 5 | Indicate if a review protocol exists, if and where it can be accessed (e.g., Web address), and, if available, provide registration information including registration number. | 6 |
| Eligibility criteria | 6 | Specify study characteristics (e.g., PICOS, length of follow-up) and report characteristics (e.g., years considered, language, publication status) used as criteria for eligibility, giving rationale. | 7-8 |
| Information sources | 7 | Describe all information sources (e.g., databases with dates of coverage, contact with study authors to identify additional studies) in the search and date last searched. | 6 |
| Search | 8 | Present full electronic search strategy for at least one database, including any limits used, such that it could be repeated. | Appendix |
| Study selection | 9 | State the process for selecting studies (i.e., screening, eligibility, included in systematic review, and, if applicable, included in the meta-analysis). | 8 |
| Data collection process | 10 | Describe method of data extraction from reports (e.g., piloted forms, independently, in duplicate) and any processes for obtaining and confirming data from investigators. | 8-9 |
| Data items | 11 | List and define all variables for which data were sought (e.g., PICOS, funding sources) and any assumptions and simplifications made. | 7-8, Appendix |
| Risk of bias in individual studies | 12 | Describe methods used for assessing risk of bias of individual studies (including specification of whether this was done at the study or outcome level), and how this information is to be used in any data synthesis. | 9 |
| Summary measures | 13 | State the principal summary measures (e.g., risk ratio, difference in means). | 9-10 |
| Synthesis of results | 14 | Describe the methods of handling data and combining results of studies, if done, including measures of consistency (e.g., I^2^) for each meta-analysis. | NA |

*From:*  Moher D, Liberati A, Tetzlaff J, Altman DG, The PRISMA Group (2009). Preferred Reporting Items for Systematic Reviews and Meta-Analyses: The PRISMA Statement. PLoS Med 6(6): e1000097. doi:10.1371/journal.pmed1000097

For more information, visit: **www.prisma-statement.org**.

**Appendix 2**

| **Search** | **Pubmed** |
| --- | --- |
| #1 | ("Randomized Controlled Trial"[Publication Type] OR "Randomized Controlled Trials as Topic"[Mesh] OR "Controlled Clinical Trial"[Publication Type] OR "Controlled Clinical Trials as Topic"[Mesh] OR "Controlled Before-After Studies"[Mesh] OR "Interrupted Time Series Analysis"[Mesh] OR Controlled Clinical Trial*[tiab] OR Randomized Controlled Trial*[tiab] OR Randomised Controlled Trial*[tiab] OR Cluster Controlled Trial*[tiab] OR Randomized Trial*[tiab] OR Randomised Trial*[tiab] OR Clinical Trial*[tiab] OR Controlled Before After[tiab] OR Interrupted Time Series[tiab]) |
| #2 | (Aged[MeSH] OR Aged[tiab] OR Elder*[tiab] OR Oldest Old[tiab] OR Sexagenarian*[tiab] OR Septuagenarian*[tiab] OR Nonagenarian*[tiab] OR Octogenarian*[tiab] OR Centenarian*[tiab] OR Supercentenarian*[tiab] OR Aging[tiab] OR Ageing[tiab] OR ((Older[tiab]) AND (People[tiab] OR Person*[tiab] OR Adult*[tiab] OR Patient[tiab] OR patients[tiab] OR Individual*[tiab] OR client*[tiab])) OR Geriatric Assessment[MeSH] OR Geriatric Assessment[tiab] OR Frailty[MeSH] OR Frailt*[tiab] OR Pulmonary Disease, Chronic Obstructive[Mesh] OR Chronic Obstructive Pulmonary Disease[tiab] OR COPD[tiab] OR bronchitis[tiab] OR emphysema[tiab] OR "Cerebrovascular Disorders"[Mesh] OR stroke[MeSH] OR stroke[tiab] OR cerebrovascular accident[tiab] OR CVA[tiab] OR Heart Diseases[Mesh] OR heart disease[tiab] OR heart failure[tiab] OR Chronic Disease[Mesh] OR chronic*[tiab] OR geriatric disease[tiab] or age-related disease[tiab] OR diabetes mellitus[Mesh] OR diabetes[tiab] OR diabetic[tiab] OR comorbidity[MeSH] OR comorbid[tiab] OR comorbidit*[tiab] OR multimorbid[tiab] OR multimorbidit*[tiab] OR Neoplasms[MeSH] OR neoplasm*[tiab] OR cancer[tiab] OR malignan*[tiab] OR tumor[tiab] OR tumors[tiab] OR Dementia[MeSH] OR dementia[tiab] OR Alzheimer Disease[Mesh] OR Alzheimer[tiab] |
| #3 | (Community Health Nurses[MeSH] OR Community Health Nursing[MeSH] OR Public Health Nursing[MeSH] OR Public Health Nurses[MeSH] OR Home Nursing[MeSH] OR Community Health Nurs*[tiab] OR Community Care Registered Nurse*[tiab] OR Community Care Nurse*[tiab] OR Public Health Nurs*[tiab] OR Home Health Nurs*[tiab] OR Community Nurs*[tiab] OR District nurs*[tiab] OR Home Nurs*[tiab] OR Visiting nurs*[tiab] OR Neighborhood Nurs*[tiab] OR Neighbourhood Nurs*[tiab] OR Home Care Nurs*[tiab] OR Homecare Nurs*[tiab] OR ((Geriatric Nursing[MeSH] OR Geriatric Nursing[tiab] OR nurs*[tiab]) AND (Home care[tiab] OR community[tiab] OR district[tiab] OR public[tiab] OR house call*[tiab] OR "House Calls"[Mesh] OR Home Care Services [MeSH] OR Home Care Service*[tiab] OR Health Visitor*[tiab]))) |
| #4 | Activities of Daily Living[MeSH] OR Activities of daily living[tiab] OR Activity of daily living[tiab] OR ADL[tiab] OR IADL[tiab] OR Mobility Limitation[MeSH] OR Mobility[tiab] OR Mobility Limitation*[tiab] OR ((Ambulation[tiab] OR Ambulatory[tiab]) AND (Difficult*[tiab])) OR Difficulty Walking[tiab] OR Accidental Falls[MeSH] OR Fall*[tiab] OR Frailty[MeSH] OR Frailt*[tiab] OR Frailness[tiab] OR Delirium[MeSH] OR Delirium[tiab] OR Weight Loss[MeSH] OR ((Weight[tiab]) AND (Loss*[tiab] OR Reduction*[tiab])) OR Pain[MeSH] OR Pain[tiab] OR Pressure Ulcer[MeSH] OR ((Pressure[tiab]) AND (Ulcer*[tiab] OR Sore*[tiab])) OR Decubitus[tiab] OR Bedsore*[tiab] OR Fatigue[MeSH] OR Fatigue[tiab] OR Dehydration[MeSH] OR Dehydration[tiab] OR Patient Compliance[MeSH] OR ((Patient[tiab] OR Treatment[tiab]) AND (Compliance[tiab] OR Adherence[tiab])) OR Anxiety[MeSH] OR Anxiet*[tiab] OR Personal Autonomy[MeSH] OR Autonomy[tiab] OR Decision Making[MeSH] OR Decision Making[tiab] OR Social Participation[tiab] OR Social Activit*[tiab] OR Caregiver Burden[tiab] OR Quality of Life[MeSH] OR Quality of Life[tiab] OR Life Quality[tiab] OR ((Emergency Medical Services[MeSH] OR Emergency Medical Service*[tiab] OR Medical Emergency Service*[tiab] OR Emergency Health Service*[tiab]) AND (Use[tiab] OR Utilization[tiab] OR Utilisation[tiab])) OR Patient Admission[MeSH] OR Patient Readmission[MeSH] OR Institutionalization[MeSH] OR Health Care Utilization[tiab] OR Healthcare Utilization[tiab] OR Admission*[tiab] OR Readmission*[tiab] OR re-admission*[tiab] OR General Practitioner Visit*[tiab] OR Nursing Home[tiab] OR Institutionalisation*[tiab] OR Institutionalization*[tiab] OR duration[tiab] OR Mortality[MeSH] OR Mortality[tiab] OR Quality of Death[tiab] OR Quality of Dying[tiab] OR "Patient Satisfaction"[Mesh] OR satisfaction[tiab] |
| #5 | #1 AND #2 AND #3 AND #4 |

| **Search** | **Cinahl** |
| --- | --- |
| #1 | ((MH "Randomized Controlled Trials+") OR (MM "Controlled Before-After Studies") OR (MM "Interrupted Time Series Analysis") OR (MM "Clinical Trials") OR TI “Controlled Clinical Trial*” OR TI “Randomized Controlled Trial*” OR TI “Randomised Controlled Trial*” OR TI “Cluster Controlled Trial*” OR TI “Randomized Trial*” OR TI “Randomised Trial*” OR TI “Clinical Trial*” OR TI “Controlled Before After” OR TI “Interrupted Time Series” OR AB “Controlled Clinical Trial*” OR AB “Randomized Controlled Trial*” OR AB “Randomised Controlled Trial*” OR AB “Cluster Controlled Trial*” OR AB “Randomized Trial*” OR AB “Randomised Trial*” OR AB “Clinical Trial*” OR AB “Controlled Before After” OR AB “Interrupted Time Series”) |
| #2 | ((MH "Aged+") OR (MH "Aged, 80 and Over+") OR TI “Aged” OR TI “Elder*” OR TI “Oldest Old” OR TI “Sexagenarian*” OR TI “Septuagenarian*” OR TI “Nonagenarian*” OR TI “Octogenarian*” OR TI “Centenarian*” OR TI “Supercentenarian*” OR TI “Aging” OR TI “Ageing” OR ((TI “Older”) AND (TI “People” OR TI “Person*” OR TI “Adult*” OR TI “Patient” OR TI “patients” OR TI “Individual*” OR TI “client*”)) OR AB “Aged” OR AB “Elder*” OR AB “Oldest Old” OR AB “Sexagenarian*” OR AB “Septuagenarian*” OR AB “Nonagenarian*” OR AB “Octogenarian*” OR AB “Centenarian*” OR AB “Supercentenarian*” OR AB “Aging” OR AB “Ageing” OR ((AB “Older”) AND (AB “People” OR AB “Person*” OR AB “Adult*” OR AB “Patient” OR AB “patients” OR AB “Individual*” OR AB “client*”)) OR (MH "Geriatric Assessment+") OR TI “geriatric assessment” OR AB “geriatric assessment” OR (MH "Frailty Syndrome") OR TI “frailt*” OR AB “frailt*” OR (MH "Lung Diseases, Obstructive+") OR (MH "Pulmonary Disease, Chronic Obstructive+") OR TI “Chronic Obstructive Pulmonary Disease” OR TI “COPD” OR AB “Chronic Obstructive Pulmonary Disease” OR AB “COPD” OR TI “bronchitis” OR TI “emphysema” OR AB “bronchitis” OR AB “emphysema” OR (MH "Cerebrovascular Disorders+") OR TI “stroke” OR TI “cerebrovascular accident” OR TI “CVA” OR AB “stroke” OR AB “cerebrovascular accident” OR AB “CVA” OR (MH "Heart Diseases+") OR TI “heart disease” OR TI “heart failure” OR AB “heart disease” OR AB “heart failure” OR (MH "Chronic Disease+") OR TI “chronic*” OR TI “geriatric disease” or TI “age-related disease” OR AB “chronic*” OR AB “geriatric disease” or AB “age-related disease” OR (MH "Diabetes Mellitus, Type 1") OR (MH "Diabetes Mellitus, Type 2") OR TI “diabetes” OR TI “diabetic” OR AB “diabetes” OR AB “diabetic” OR (MH "Comorbidity") OR TI “comorbid” OR TI “comorbidit*” OR TI “multimorbid” OR TI “multimorbidit*” OR AB “comorbid” OR AB “comorbidit*” OR AB “multimorbid” OR AB “multimorbidit*” OR (MH "Neoplasms") OR TI “neoplasm*” OR TI “cancer” OR TI “malignan*” OR TI “tumor” OR TI “tumors” OR AB “neoplasm*” OR AB “cancer” OR AB “malignan*” OR AB “tumor” OR AB “tumors” OR (MH "Dementia+") OR TI “dementia” OR AB “dementia” OR (MH "Alzheimer's Disease") OR TI “Alzheimer” OR AB “Alzheimer”) |
| #3 | ((MH "nurses, community health+") OR (MH "community health nursing+") OR (MH "public health nursing+") OR (MH "nurses, public health+") OR (MH "home nursing+") OR TI "community health nurs*" OR AB "community health nurs*" OR TI "community care registered nurse#" OR AB "community care registered nurse#" OR TI "community care nurse#" OR AB "community care nurse#" OR TI "public health nurs*" OR AB "public health nurs*" OR TI "home health nurs*" OR AB "home health nurs*" OR TI "community nurs*" OR AB "community nurs*" OR TI "district nurs*" OR AB "district nurs*" OR TI "home nurs*" OR AB "home nurs*" OR TI "visiting nurs*" OR AB "visiting nurs*" OR TI "neighborhood nurs*" OR AB "neighborhood nurs*" OR TI "neighbourhood nurs*" OR AB "neighbourhood nurs*" OR TI "home care nurs*" OR AB "home care nurs*" OR TI "homecare nurs*" OR AB "homecare nurs*" OR ((MH "Geriatric Nursing+") OR TI "Geriatric Nursing" OR AB "Geriatric Nursing" OR TI “nurs*” OR AB “nurs*”) AND (TI “home care” OR AB “home care” OR TI “community” OR AB “community” OR TI “district” OR AB “district” OR TI “public” OR AB “public” OR TI “house call*” OR AB “house call*” OR (MH "house calls+") OR (MH "Home Care Services+") OR TI "Home Care Service*" OR AB "Home Care Service*" OR TI "health visitor*" OR AB "health visitor*"))) |
| #4 | ((MH "Activities of Daily Living+") OR (MH "Self-Care (Iowa NOC)+") OR (MH "Self-Care Deficit (Saba CCC)+") OR TI “Activities of daily living” OR TI “Activity of daily living” OR TI “ADL” OR TI “IADL” OR AB “Activities of daily living” OR AB “Activity of daily living” OR AB “ADL” OR AB “IADL” OR MH "Impaired Physical Mobility (NANDA)+") OR (MH "Mobility (Iowa NOC)+") OR TI “Mobility” OR TI “Mobility Limitation*” OR (( TI “Ambulation” OR TI “Ambulatory”) AND (TI “Difficult*”)) OR TI “Difficulty Walking” OR AB “Mobility” OR AB “Mobility Limitation*” OR (( AB “Ambulation” OR AB “Ambulatory”) AND (AB “Difficult*”)) OR AB “Difficulty Walking” OR (MH "Accidental Falls") OR TI “fall*” OR AB “fall*” OR (MH "Frailty Syndrome") OR TI “Frailt*” OR TI “Frailness” OR AB “Frailt*” OR AB “Frailness” OR (MH "Delirium") OR TI “delirium” OR AB “delirium” OR (MH "Weight Loss+") OR ((TI “Weight”) AND (TI “Loss*” OR TI “Reduction*”)) OR ((AB “Weight”) AND (AB “Loss*” OR AB “Reduction*”)) OR (MH "Pain+") OR TI “pain” OR AB “pain” OR (MH "Skin Ulcer+") OR ((TI “Pressure”) AND (TI “Ulcer*” OR TI “Sore*”)) OR TI “Decubitus” OR TI “Bedsore*” OR ((AB “Pressure”) AND (AB “Ulcer*” OR AB “Sore*”)) OR AB “Decubitus” OR AB “Bedsore*” OR (MH "Fatigue+") OR TI “fatigue” OR AB “fatigue” OR (MH "Dehydration") OR TI “dehydration” OR AB “dehydration” OR (MH "Patient Compliance+") OR ((TI “Patient” OR TI “Treatment”) AND (TI “Compliance” OR TI “Adherence”)) OR ((AB “Patient” OR AB “Treatment”) AND (AB “Compliance” OR AB “Adherence”)) OR (MH "Anxiety+") OR TI “anxiet*” OR AB “anxiet*” OR (MH "Patient Autonomy") OR TI “Autonomy” OR AB “Autonomy” OR (MH "Decision Making+") OR TI “Decision Making” OR TI “Social Participation” OR TI “Social Activit*” OR AB “Decision Making” OR AB “Social Participation” OR AB “Social Activit*” OR (MH "Caregiver Burden") OR TI “Caregiver Burden” OR AB “Caregiver Burden” OR (MH "Quality of Life+") OR TI “Quality of Life” OR TI “Life Quality” OR AB “Quality of Life” OR AB “Life Quality” OR (((MH "Emergency Medical Services+") OR TI “Emergency Medical Service*” OR TI “Medical Emergency Service*” OR TI “Emergency Health Service*” OR AB “Emergency Medical Service*” OR AB “Medical Emergency Service*” OR AB “Emergency Health Service*”) AND (TI “Use” OR TI “Utilization” OR TI “Utilisation” OR AB “Use” OR AB “Utilization” OR AB “Utilisation”)) OR (MH "Institutionalization+") OR TI “Health Care Utilization” OR TI “Healthcare Utilization” OR TI “Admission*” OR TI “Readmission*” OR TI “re-admission*” OR TI “General Practitioner Visit*” OR TI “Nursing Home” OR TI “Institutionalisation*” OR TI “Institutionalization*” OR TI “duration” OR AB “Health Care Utilization” OR AB “Healthcare Utilization” OR AB “Admission*” OR AB “Readmission*” OR AB “re-admission*” OR AB “General Practitioner Visit*” OR AB “Nursing Home” OR AB “Institutionalisation*” OR AB “Institutionalization*” OR AB “duration” OR (MH "Mortality+") OR TI “Mortality” OR TI “Quality of Death” OR TI “Quality of Dying” OR AB “Mortality” OR AB “Quality of Death” OR AB “Quality of Dying” OR (MH "Patient Satisfaction+") OR TI “satisfaction” OR AB “satisfaction” ) |
| #5 | #1 AND #2 AND #3 AND #4 |

| **Search** | **PSYCH INFO** |
| --- | --- |
| #1 | ((DE "Randomized Controlled Trials") OR (DE "Randomized Clinical Trials") OR (DE "Clinical Trials") OR TI “Controlled Clinical Trial*” OR TI “Randomized Controlled Trial*” OR TI “Randomised Controlled Trial*” OR TI “Cluster Controlled Trial*” OR TI “Randomized Trial*” OR TI “Randomised Trial*” OR TI “Clinical Trial*” OR TI “Controlled Before After” OR TI “Interrupted Time Series” OR AB “Controlled Clinical Trial*” OR AB “Randomized Controlled Trial*” OR AB “Randomised Controlled Trial*” OR AB “Cluster Controlled Trial*” OR AB “Randomized Trial*” OR AB “Randomised Trial*” OR AB “Clinical Trial*” OR AB “Controlled Before After” OR AB “Interrupted Time Series”) |
| #2 | ((DE "Aging") OR TI “Aged” OR TI “Elder*” OR TI “Oldest Old” OR TI “Sexagenarian*” OR TI “Septuagenarian*” OR TI “Nonagenarian*” OR TI “Octogenarian*” OR TI “Centenarian*” OR TI “Supercentenarian*” OR TI “Aging” OR TI “Ageing” OR ((TI “Older”) AND (TI “People” OR TI “Person*” OR TI “Adult*” OR TI “Patient” OR TI “patients” OR TI “Individual*” OR TI “client*”)) OR AB “Aged” OR AB “Elder*” OR AB “Oldest Old” OR AB “Sexagenarian*” OR AB “Septuagenarian*” OR AB “Nonagenarian*” OR AB “Octogenarian*” OR AB “Centenarian*” OR AB “Supercentenarian*” OR AB “Aging” OR AB “Ageing” OR ((AB “Older”) AND (AB “People” OR AB “Person*” OR AB “Adult*” OR AB “Patient” OR AB “patients” OR AB “Individual*” OR AB “client*”)) OR (DE "Geriatric Assessment") OR TI “geriatric assessment” OR AB “geriatric assessment” OR (DE "Health Impairments" OR DE "Homebound") OR TI “frailt*” OR AB “frailt*” OR DE "Lung Disorders" OR DE "Chronic Obstructive Pulmonary Disease" OR DE "Cystic Fibrosis" OR DE "Pneumonia" OR DE "Pulmonary Emphysema" OR DE "Pulmonary Tuberculosis" OR TI “Chronic Obstructive Pulmonary Disease” OR TI “COPD” OR AB “Chronic Obstructive Pulmonary Disease” OR AB “COPD” OR TI “bronchitis” OR TI “emphysema” OR AB “bronchitis” OR AB “emphysema” OR DE "Cerebrovascular Disorders" OR DE "Cerebral Arteriosclerosis" OR DE "Cerebral Hemorrhage" OR DE "Cerebral Ischemia" OR DE "Cerebral Small Vessel Disease" OR DE "Cerebrovascular Accidents" OR DE "Subarachnoid Hemorrhage" OR TI “stroke” OR TI “cerebrovascular accident” OR TI “CVA” OR AB “stroke” OR AB “cerebrovascular accident” OR AB “CVA” OR DE "Heart Disorders" OR DE "Angina Pectoris" OR DE "Arrhythmias (Heart)" OR DE "Coronary Thromboses" OR DE "Myocardial Infarctions" OR TI “heart disease” OR TI “heart failure” OR AB “heart disease” OR AB “heart failure” OR DE "Chronic Illness" OR DE "Chronic Alcoholic Intoxication" OR DE "Chronic Fatigue Syndrome" OR DE "Chronic Mental Illness" OR DE "Chronic Pain" OR DE "Chronically Ill Children" OR TI “chronic*” OR TI “geriatric disease” or TI “age-related disease” OR AB “chronic*” OR AB “geriatric disease” or AB “age-related disease” OR DE "Diabetes" OR DE "Diabetes Insipidus" OR DE "Diabetes Mellitus" OR DE "Type 2 Diabetes" OR DE "Diabetes Mellitus" OR DE "Gestational Diabetes" OR DE "Type 2 Diabetes" OR DE "Type 2 Diabetes" OR DE "Blood Sugar" OR TI “diabetes” OR TI “diabetic” OR AB “diabetes” OR AB “diabetic” OR DE "Comorbidity" OR TI “comorbid” OR TI “comorbidit*” OR TI “multimorbid” OR TI “multimorbidit*” OR AB “comorbid” OR AB “comorbidit*” OR AB “multimorbid” OR AB “multimorbidit*” OR DE "Neoplasms" OR DE "Benign Neoplasms" OR DE "Breast Neoplasms" OR DE "Endocrine Neoplasms" OR DE "Leukemias" OR DE "Melanoma" OR DE "Metastasis" OR DE "Nervous System Neoplasms" OR DE "Terminal Cancer" OR TI “neoplasm*” OR TI “cancer” OR TI “malignan*” OR TI “tumor” OR TI “tumors” OR AB “neoplasm*” OR AB “cancer” OR AB “malignan*” OR AB “tumor” OR AB “tumors” OR DE "Dementia" OR DE "AIDS Dementia Complex" OR DE "Dementia with Lewy Bodies" OR DE "Presenile Dementia" OR DE "Pseudodementia" OR DE "Semantic Dementia" OR DE "Senile Dementia" OR DE "Vascular Dementia" OR TI “dementia” OR AB “dementia” OR DE "Alzheimer's Disease" OR TI “Alzheimer” OR AB “Alzheimer”) |
| #3 | (DE "Home Care Personnel" OR DE "Public Health Service Nurses" OR DE "Community Services" OR DE "Community Mental Health Services" OR DE "Community Welfare Services" OR DE "Emergency Services" OR DE "Home Care" OR DE "Home Visiting Programs" OR DE "Public Health Services" OR DE "Community Mental Health Services" OR DE "Community Counseling" OR DE "Public Health Services" OR DE "Public Service Announcements" OR TI "community health nurs*" OR AB "community health nurs*" OR TI "community care registered nurse#" OR AB "community care registered nurse#" OR TI "community care nurse#" OR AB "community care nurse#" OR TI "public health nurs*" OR AB "public health nurs*" OR TI "home health nurs*" OR AB "home health nurs*" OR TI "community nurs*" OR AB "community nurs*" OR TI "district nurs*" OR AB "district nurs*" OR TI "home nurs*" OR AB "home nurs*" OR TI "visiting nurs*" OR AB "visiting nurs*" OR TI "neighborhood nurs*" OR AB "neighborhood nurs*" OR TI "neighbourhood nurs*" OR AB "neighbourhood nurs*" OR TI "home care nurs*" OR AB "home care nurs*" OR TI "homecare nurs*" OR AB "homecare nurs*" OR ((TI "Geriatric Nursing" OR AB "Geriatric Nursing" OR TI “nurs*” OR AB “nurs*”) AND (TI “home care” OR AB “home care” OR TI “community” OR AB “community” OR TI “district” OR AB “district” OR TI “public” OR AB “public” OR TI “house call*” OR AB “house call*” OR TI "Home Care Service*" OR AB "Home Care Service*" OR TI "health visitor*" OR AB "health visitor*"))) |
| #4 | (DE "Activities of Daily Living" OR DE "Functional Status" OR TI “Activities of daily living” OR TI “Activity of daily living” OR TI “ADL” OR TI “IADL” OR AB “Activities of daily living” OR AB “Activity of daily living” OR AB “ADL” OR AB “IADL” OR DE "Physical Mobility" OR TI “Mobility” OR TI “Mobility Limitation*” OR (( TI “Ambulation” OR TI “Ambulatory”) AND (TI “Difficult*”)) OR TI “Difficulty Walking” OR AB “Mobility” OR AB “Mobility Limitation*” OR (( AB “Ambulation” OR AB “Ambulatory”) AND (AB “Difficult*”)) OR AB “Difficulty Walking” OR DE "Falls" OR TI “fall*” OR AB “fall*” OR TI “Frailt*” OR TI “Frailness” OR AB “Frailt*” OR AB “Frailness” OR DE "Delirium" OR TI “delirium” OR AB “delirium” OR DE "Weight Loss" OR ((TI “Weight”) AND (TI “Loss*” OR TI “Reduction*”)) OR ((AB “Weight”) AND (AB “Loss*” OR AB “Reduction*”)) OR DE "Pain" OR DE "Aphagia" OR DE "Back Pain" OR DE "Chronic Pain" OR DE "Headache" OR DE "Myofascial Pain" OR DE "Neuralgia" OR DE "Neuropathic Pain" OR DE "Somatoform Pain Disorder" OR TI “pain” OR AB “pain” OR ((TI “Pressure”) AND (TI “Ulcer*” OR TI “Sore*”)) OR TI “Decubitus” OR TI “Bedsore*” OR ((AB “Pressure”) AND (AB “Ulcer*” OR AB “Sore*”)) OR AB “Decubitus” OR AB “Bedsore*” OR DE "Fatigue" OR TI “fatigue” OR AB “fatigue” OR DE "Dehydration" OR TI “dehydration” OR AB “dehydration” OR DE "Compliance" OR DE "Treatment Compliance" OR ((TI “Patient” OR TI “Treatment”) AND (TI “Compliance” OR TI “Adherence”)) OR ((AB “Patient” OR AB “Treatment”) AND (AB “Compliance” OR AB “Adherence”)) OR DE "Anxiety Disorders" OR DE "Castration Anxiety" OR DE "Death Anxiety" OR DE "Generalized Anxiety Disorder" OR DE "Obsessive Compulsive Disorder" OR DE "Panic Attack" OR DE "Panic Disorder" OR DE "Phobias" OR DE "Separation Anxiety Disorder" OR DE "Trichotillomania" OR TI “anxiet*” OR AB “anxiet*” OR DE "Autonomy" OR DE "Autonomy (Government)" OR DE "Empowerment" OR DE "Independence (Personality)" OR TI “Autonomy” OR AB “Autonomy” OR DE "Decision Making" OR DE "Choice Behavior" OR DE "Group Decision Making" OR TI “Decision Making” OR TI “Social Participation” OR TI “Social Activit*” OR AB “Decision Making” OR AB “Social Participation” OR AB “Social Activit*” OR DE "Participation" OR DE "Athletic Participation" OR DE "Client Participation" OR DE "Group Participation" OR DE "Participative Management" OR DE "Caregiver Burden" OR TI “Caregiver Burden” OR AB “Caregiver Burden” OR DE "Quality of Life" OR DE "Health Related Quality of Life" OR DE "Quality of Work Life" OR TI “Quality of Life” OR TI “Life Quality” OR AB “Quality of Life” OR AB “Life Quality” OR ((DE "Emergency Services" OR DE "Crisis Intervention Services" OR TI “Emergency Medical Service*” OR TI “Medical Emergency Service*” OR TI “Emergency Health Service*” OR AB “Emergency Medical Service*” OR AB “Medical Emergency Service*” OR AB “Emergency Health Service*”) AND (TI “Use” OR TI “Utilization” OR TI “Utilisation” OR AB “Use” OR AB “Utilization” OR AB “Utilisation”)) OR (DE "Institutionalization") OR (DE "Institutionalization" OR DE "Hospitalization" OR DE "Incarceration" OR DE "Institution Visitation" OR DE "Institutional Release") OR DE "Hospital Admission" OR DE "Psychiatric Hospital Admission" OR TI “Health Care Utilization” OR TI “Healthcare Utilization” OR TI “Admission*” OR TI “Readmission*” OR TI “re-admission*” OR TI “General Practitioner Visit*” OR TI “Nursing Home” OR TI “Institutionalisation*” OR TI “Institutionalization*” OR TI “duration” OR AB “Health Care Utilization” OR AB “Healthcare Utilization” OR AB “Admission*” OR AB “Readmission*” OR AB “re-admission*” OR AB “General Practitioner Visit*” OR AB “Nursing Home” OR AB “Institutionalisation*” OR AB “Institutionalization*” OR AB “duration” OR DE "Death and Dying" OR DE "Assisted Suicide" OR DE "Child Death" OR DE "Euthanasia" OR DE "Mortality Rate" OR DE "Mortality Risk" OR DE "Parental Death" OR DE "Partner Death" OR DE "Sudden Death" OR TI “Mortality” OR TI “Quality of Death” OR TI “Quality of Dying” OR AB “Mortality” OR AB “Quality of Death” OR AB “Quality of Dying” OR MM "Client Satisfaction" OR TI "Client Satisfaction" OR AB "Client Satisfaction") |
| #5 | #1 AND #2 AND #3 AND #4 |

| **Search** | **Embase** |
| --- | --- |
| #1 | ('randomized controlled trial'/exp OR 'controlled clinical trial'/exp OR ‘Controlled Clinical Trial*’:ti,ab,kw OR ‘Randomized Controlled Trial*’:ti,ab,kw OR ‘Randomised Controlled Trial*’:ti,ab,kw OR ‘Cluster Controlled Trial*’:ti,ab,kw OR ‘Randomized Trial*’:ti,ab,kw OR ‘Randomised Trial*’:ti,ab,kw OR ‘Clinical Trial*’:ti,ab,kw OR ‘Controlled Before After’:ti,ab,kw OR ‘Interrupted Time Series’:ti,ab,kw) |
| #2 | ('aged'/exp OR ‘Aged’:ti,ab,kw OR ‘Elder*’:ti,ab,kw OR ‘Oldest Old’:ti,ab,kw OR ‘Sexagenarian*’:ti,ab,kw OR ‘Septuagenarian*’:ti,ab,kw OR ‘Nonagenarian*’:ti,ab,kw OR ‘Octogenarian*’:ti,ab,kw OR ‘Centenarian*’:ti,ab,kw OR ‘Supercentenarian*’:ti,ab,kw OR ‘Aging’:ti,ab,kw OR ‘Ageing’:ti,ab,kw OR ((‘Older’:ti,ab,kw) AND (‘People’:ti,ab,kw OR ‘Person*’:ti,ab,kw OR ‘Adult*’:ti,ab,kw OR ‘Patient’:ti,ab,kw OR ‘patients’:ti,ab,kw OR ‘Individual*’:ti,ab,kw OR ‘client*’:ti,ab,kw)) OR 'geriatric assessment'/exp OR ‘Geriatric Assessment’:ti,ab,kw OR 'frailty'/exp OR ‘Frailt*’:ti,ab,kw OR 'lung disease'/exp OR 'obstructive airway disease'/exp OR 'chronic obstructive lung disease'/exp OR ‘Chronic Obstructive Pulmonary Disease’:ti,ab,kw OR ‘COPD’:ti,ab,kw OR ‘bronchitis’:ti,ab,kw OR ‘emphysema’:ti,ab,kw OR 'cerebrovascular accident'/exp OR ‘stroke’:ti,ab,kw OR ‘cerebrovascular accident’:ti,ab,kw OR ‘CVA’:ti,ab,kw OR 'heart disease'/exp OR ‘heart disease’:ti,ab,kw OR ‘heart failure’:ti,ab,kw OR 'chronic disease'/exp OR ‘chronic*’:ti,ab,kw OR ‘geriatric disease’:ti,ab,kw or ‘age-related disease’:ti,ab,kw OR 'diabetes mellitus'/de OR ‘diabetes’:ti,ab,kw OR ‘diabetic’:ti,ab,kw OR 'comorbidity'/exp OR 'multiple chronic conditions'/exp OR ‘comorbid’:ti,ab,kw OR ‘comorbidit*’:ti,ab,kw OR ‘multimorbid’:ti,ab,kw OR ‘multimorbidit*’:ti,ab,kw OR 'malignant neoplasm'/exp OR ‘neoplasm*’:ti,ab,kw OR ‘cancer’:ti,ab,kw OR ‘malignan*’:ti,ab,kw OR ‘tumor’:ti,ab,kw OR ‘tumors’:ti,ab,kw OR 'dementia'/exp OR ‘dementia’:ti,ab,kw OR Alzheimer.ti,ab,kw) |
| #3 | ('community health nursing'/exp OR ‘Community Health Nurs*’:ti,ab,kw OR ‘Community Care Registered Nurse*’:ti,ab,kw OR ‘Community Care Nurse*’:ti,ab,kw OR ‘Public Health Nurs*’:ti,ab,kw OR ‘Home Health Nurs*’:ti,ab,kw OR ‘Community Nurs*’:ti,ab,kw OR ‘District nurs*’:ti,ab,kw OR ‘Home Nurs*’:ti,ab,kw OR ‘Visiting nurs*’:ti,ab,kw OR ‘Neighborhood Nurs*’:ti,ab,kw OR ‘Neighbourhood Nurs*’:ti,ab,kw OR ‘Home Care Nurs*’:ti,ab,kw OR ‘Homecare Nurs*’:ti,ab,kw OR (('geriatric nursing'/exp OR ‘Geriatric Nursing’:ti,ab,kw OR ‘nurs*’:ti,ab,kw) AND (‘Home care’:ti,ab,kw OR ‘community’:ti,ab,kw OR ‘district’:ti,ab,kw OR ‘public’:ti,ab,kw OR ‘house call*’:ti,ab,kw OR 'home visit'/exp OR 'home care'/exp OR ‘Home Care Service*’:ti,ab,kw OR ‘Health Visitor*’:ti,ab,kw))) |
| #4 | ('daily life activity'/exp OR 'instrumental activities of daily living'/exp OR ‘Activities of daily living’:ti,ab,kw OR ‘Activity of daily living’:ti,ab,kw OR ‘ADL’:ti,ab,kw OR ‘IADL’:ti,ab,kw OR 'patient mobility'/exp OR 'disability'/exp OR ‘Mobility’:ti,ab,kw OR ‘Mobility Limitation*’:ti,ab,kw OR ((‘Ambulation’:ti,ab,kw OR ‘Ambulatory’:ti,ab,kw) AND (‘Difficult*’:ti,ab,kw)) OR ‘Difficulty Walking’:ti,ab,kw OR 'falling'/exp OR 'falls'/exp OR ‘Fall*’:ti,ab,kw OR 'frailty'/exp OR ‘Frailt*’:ti,ab,kw OR ‘Frailness’:ti,ab,kw OR 'delirium'/exp OR ‘Delirium’:ti,ab,kw OR 'body weight loss'/exp OR ((‘Weight’:ti,ab,kw) AND (‘Loss*’:ti,ab,kw OR ‘Reduction*’:ti,ab,kw)) OR 'pain'/exp OR ‘Pain’:ti,ab,kw OR 'skin ulcer'/exp OR ((‘Pressure’:ti,ab,kw) AND (‘Ulcer*’:ti,ab,kw OR ‘Sore*’:ti,ab,kw)) OR ‘Decubitus’:ti,ab,kw OR ‘Bedsore*’:ti,ab,kw OR 'fatigue'/exp OR ‘Fatigue’:ti,ab,kw OR 'dehydration'/exp OR ‘Dehydration’:ti,ab,kw OR 'patient compliance'/exp OR ((‘Patient’:ti,ab,kw OR ‘Treatment’:ti,ab,kw) AND (‘Compliance’:ti,ab,kw OR ‘Adherence’:ti,ab,kw)) OR 'anxiety disorder'/exp OR ‘Anxiet*’:ti,ab,kw OR 'personal autonomy'/exp OR 'autonomy'/exp OR ‘Autonomy’:ti,ab,kw OR 'decision making'/exp OR ‘Decision Making’:ti,ab,kw OR ‘Social Participation’:ti,ab,kw OR ‘Social Activit*’:ti,ab,kw OR 'caregiver burden'/exp OR ‘Caregiver Burden’:ti,ab,kw OR 'quality of life'/exp OR ‘Quality of Life’:ti,ab,kw OR ‘Life Quality’:ti,ab,kw OR (('emergency health service'/exp OR ‘Emergency Medical Service*’:ti,ab,kw OR ‘Medical Emergency Service*’:ti,ab,kw OR ‘Emergency Health Service*’:ti,ab,kw) AND (‘Use’:ti,ab,kw OR ‘Utilization’:ti,ab,kw OR ‘Utilisation’:ti,ab,kw)) OR 'hospital admission'/exp OR 'hospital readmission'/exp OR 'institutional care'/exp OR ‘Health Care Utilization’:ti,ab,kw OR ‘Healthcare Utilization’:ti,ab,kw OR ‘Admission*’:ti,ab,kw OR ‘Readmission*’:ti,ab,kw OR ‘re-admission*’:ti,ab,kw OR ‘General Practitioner Visit*’:ti,ab,kw OR ‘Nursing Home’:ti,ab,kw OR ‘Institutionalisation*’:ti,ab,kw OR ‘Institutionalization*’:ti,ab,kw OR ‘duration’:ti,ab,kw OR 'mortality'/exp OR ‘Mortality’:ti,ab,kw OR ‘Quality of Death’:ti,ab,kw OR ‘Quality of Dying’:ti,ab,kw OR 'patient satisfaction'/exp OR 'patient satisfaction':ti,ab,kw) |
| #5 | #1 AND #2 AND #3 AND #4 |

**Appendix 3**

|  | **Criteria** |
| --- | --- |
| **Population: older people** | **INCLUDE** Mean age > 60 years  **EXCLUDE** Caregivers |
| **Intervention: care delivered by nurses** | **INCLUDE** intervention is defined as “any treatment based upon clinical judgment and knowledge that a nurse performs to enhance patient outcomes” (NIC).  **INCLUDE** care delivered by a district nurse. Synonyms for district nurse include community health nurse, community nurse, home care nurse or home nurse.  **INCLUDE** intervention with at least one face-to-face moment with the patient. This can be at home or in the hospital **only if** it is transitional care (hospital to home). Face to face can be conducted using telehealth.  **INCLUDE** interventions delivered by nurses, nurse-led care, nurse coordinated care or care largely delivered by nurses  **INCLUDE** multidisciplinary interventions **only if** at least 50% of the intervention is delivered by the district nurse.  **EXCLUDE** care delivered by a nurse working from the General Practitioner (e.g. nurse practitioner, advanced practice nurse (APN))  **EXCLUDE** care delivered by a nurse working from the hospital (e.g. liaison nurse, specialized nurse).  **EXCLUDE** if it is unclear if the nurse providing the intervention is a district nurse.  **EXCLUDE** Studies focusing on educational interventions directed solely at other healthcare providers |
| **Comparator** | **INCLUDE** all comparators |
| **Outcomes** | **INCLUDE** At least one of the used outcomes should be nurse-sensitive for district nursing care: Activities for daily living (ADL), Mobility, Falls, Frailty, Pain, Decubitus, Fatigue, Unintentional weight loss, Dehydration, Anxiety, Compliance, Delirium, Autonomy, Decision making, Participation with social activities, Burden informal caregiver, Quality of life, Satisfaction with district nursing care, Meaningful life, Unplanned hospital admission, Unplanned hospital readmission, Emergency department or service use, Duration of district nursing, Intensity of district nursing, Place of death, Quality of dying and death. |
| **Setting** | **INCLUDE** home and community care in a primary care setting.  **EXCLUDE** hospital setting  **EXCLUDE** general practitioner setting  **EXCLUDE** if the setting is unclear |
| **Design** | **INCLUDE** randomized controlled trials (RCTs) (including small RCTs like pilot studies, as long as they used randomization), controlled clinical trials (CCT), controlled before-and-after studies (CBAs) and interrupted time series studies (ITS)  **EXCLUDE** secondary data analyses using trial data when it does not focus on nursing interventions.  **EXCLUDE** Studies with only observational data used to describe the work of community care nurses.  **EXCLUDE** quasi-experimental design without randomization  **EXCLUDE** Qualitative study designs.  **EXCLUDE** Process evaluation, study protocols  **EXCLUDE** Systematic review |
| **Language** | **INCLUDE** articles in English or Dutch |
| **Publication date** | **INCLUDE** all publication dates |
| **Country** | **INCLUDE** all countries |

**Appendix 4**

| **Outcomes identified in the systematic review** | **Is the outcome included in the Delphi study by Veldhuizen et al.?** | **Is the outcome assessed as nurse-sensitive in the Delphi study?** |
| --- | --- | --- |
| **Functional health** |  |  |
| **Activities of daily living, disability, impairment in mobility, self-care agency** | Yes: ADL, mobility | Yes, nurse-sensitive |
| **Functional status** | Yes: ADL; mobility | Yes, nurse-sensitive |
| **Gait and balance** | Yes: mobility | Yes, nurse-sensitive |
| **Self-care adherence** | Yes: ADL, compliance | Yes, nurse-sensitive |
| **Handicap** | No | NA |
| **Physiologic health** |  |  |
| **Cognitive functioning** | Yes: cognitive functioning | Unclear |
| **Number of medications** | Yes: polypharmacy | Not nurse-sensitive |
| **Potentially inappropriate medications, excessive use of psychotropic, anticholinergic and serotonergic load, drug-drug interactions** | No | NA |
| **Blood pressure (systolic and diastolic)** | No | NA |
| **Episodes of urine loss** | Yes: bladder continence, | Unclear |
| **Micturition frequency** | Yes: bladder continence, | Unclear |
| **Urine loss severity in grams** | Yes: bladder continence, | Unclear |
| **Psychosocial health** |  |  |
| **Mental health, emotional well-being, psychological state** | Yes: signs of depression, anxiety | Signs of depression: unclear  Anxiety: Yes, nurse-sensitive |
| **Depressive complaints, affect** | Yes: signs of depression | Unclear |
| **Loneliness** | Yes: loneliness | Unclear |
| **Social support** | No | NA |
| **Social functioning** | Yes: participation in social activities | Yes, nurse-sensitive |
| **Self-esteem** | No | NA |
| **Coping style** | No | NA |
| **Morale** | No | NA |
| **Health knowledge and behaviour** |  |  |
| **Knowledge about aspects of disease and about contact with local community, desire for information** | Yes: knowledge of the patient | Not nurse-sensitive |
| **Self-efficacy, locus of control, locus of authority in decision making** | Yes: autonomy | Yes, nurse-sensitive |
| **Number of falls** | Yes: falls | Yes, nurse-sensitive |
| **Concerns about falls and avoidance of activity, fear of falling** | Yes: falls | Yes, nurse-sensitive |
| **Health behavior** | Yes: compliance, problem behavior | Yes, nurse-sensitive |
| **Independence to manage health** | Yes: autonomy, decision making | Yes, nurse-sensitive |
| **Perceived ability to manage health** | Yes: autonomy decision making | Yes, nurse-sensitive |
| **Perceived health** |  |  |
| **General health (self-rated)** | No | NA |
| **Quality of life** | Yes: quality of life, meaningful life, quality of dying and death | Yes, nurse-sensitive |
| **Satisfaction with care provided** | Yes: satisfaction with district nursing care | Yes, nurse-sensitive |
| **Symptom intensity/burden, health complaints, physical complaints** | No in general, but decubitus, dehydration, fatigue, pain, and weight loss were measured | decubitus, dehydration, fatigue, pain, and weight loss were measured: Yes, nurse-sensitive |
| **Health problems, changes in self-reported problems,** | No in general, but decubitus, dehydration, fatigue, pain, and weight loss were measured | decubitus, dehydration, fatigue, pain, and weight loss were measured: Yes, nurse-sensitive |
| **Quality adjusted life years (QALY)** | No | NA |
| **Family health** |  |  |
| **Caregiver burden** | Yes: informal caregiver burden | Yes, nurse-sensitive |
| **Death** |  |  |
| **Mortality status, time until death, mortality rate, mortality** | Yes: Death | No, not nurse-sensitive |
| **Healthcare utilization** |  |  |
| **Health care utilization: (time to) hospital readmission (in days)** | Yes, unplanned hospital (re)admission. | Yes, nurse-sensitive |
| **Healthcare utilization: (time to) community nursing** | Yes: duration and intensity of district nursing care | Yes, nurse-sensitive |
| **Health care utilization: (time to) institutionalization to nursing home / care home** | Yes, nursing home admission | Unclear |
| **Healthcare utilization: physician visits during and after hours** | Yes, general practitioner visit | Unclear |
| **Healthcare utilization: emergency care attendance** | Yes: emergency department or service use | Yes, nurse-sensitive |
| **Health care utilization: number of days in hospital wards, hospital stay** | No | NA |
| **Health care utilization: outpatient clinics** | No | NA |
| **Healthcare utilization: physiotherapy contacts** | No | NA |
| **Aids and modifications to the home** | No | NA |
